# An Alignment-Based Implementation of a Holistic Ontology Integration Method^☆^

**DOI:** 10.1016/j.mex.2021.101460

**Published:** 2021-07-23

**Authors:** Inès Osman, Salvatore Flavio Pileggi, Sadok Ben Yahia, Gayo Diallo

**Affiliations:** a LIPAH - LR11ES14, Faculty of Sciences of Tunis, University of Tunis El Manar, Tunis 2092, Tunisia; b School of Information, Systems and Modelling, University of Technology Sydney, Australia; c Department of Software Science, Tallinn University of Technology, Estonia; d INRIA SISTM, Team ERIAS - INSERM Bordeaux Population Health Research Center, University of Bordeaux, F-33000 Bordeaux, France

**Keywords:** Ontology, Ontology integration, Ontology merging, Ontology refactoring, Semantic interoperability, Bridge ontology, Full-Merge ontology, 00-01, 99-00

## Abstract

Despite the intense research activity in the last two decades, ontology integration still presents a number of challenging issues. As ontologies are continuously growing in number, complexity and size and are adopted within open distributed systems such as the Semantic Web, integration becomes a central problem and has to be addressed in a context of increasing scale and heterogeneity. In this paper, we describe a holistic alignment-based method for customized ontology integration. The holistic approach proposes additional challenges as multiple ontologies are jointly integrated at once, in contrast to most common approaches that perform an incremental pairwise ontology integration. By applying consolidated techniques for ontology matching, we investigate the impact on the resulting ontology. The proposed method takes multiple ontologies as well as pairwise alignments and returns a refactored/non-refactored integrated ontology that faithfully preserves the original knowledge of the input ontologies and alignments. We have tested the method on large biomedical ontologies from the LargeBio OAEI track. Results show effectiveness, and overall, a decreased integration cost over multiple ontologies.

• OIAR and AROM are two implementations of the proposed method.
• OIAR creates a bridge ontology, and AROM creates a fully merged ontology.
• The implementation includes the option of ontology refactoring.

## Graphical abstract

**Figure:**
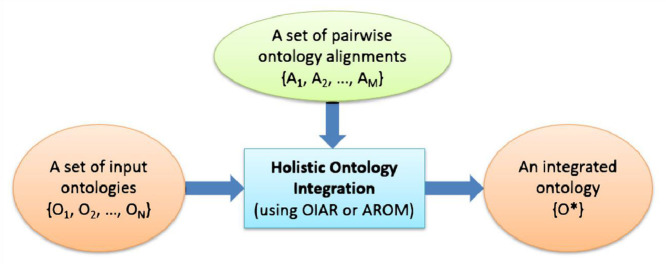

Specifications Table

**Table:** 

Subject Area:	Computer Science
More specific subject area:	Knowledge Engineering
Method name:	Holistic integration of multiple ontologies using alignments between ontology pairs (OIAR/AROM)
Name and reference of original method:	N.A.
Resource availability:	OWL language + JAVA language + OWL~API + Alignment API + IDE (Eclipse, IntelliJ, or NetBeans) + ELK reasoner + OAEI tracks

## 1 Introduction

Ontology has become a more and more popular concept in Computer Science to represent and share knowledge within digital environments. Such a rich data model provides a common understanding of a given domain by defining a shared vocabulary which is formally specified in a machine-processable format [2].

However, in open and distributed systems such as the Semantic Web [3], [4], heterogeneity still cannot be avoided. In recent years, ontology-based approaches have been adopted in the context of many domains, as well as across different domains according to a multi/trans-disciplinary philosophy. Due to this disconnected development of ontologies, many ontologies in identical, similar, complementary or interdisciplinary domains have been developed. As a result, applications or information systems, relying on these ontologies, cannot achieve communication nor interoperability. Ontology integration addresses this issue by creating a new ontology that groups the knowledge contained in different existing ontologies and that can be therefore used by different heterogeneous applications.

Nowadays, the ontology community has adopted the idea of splitting the ontology integration problem into *matching* and *merging* sub-tasks, where *matching* is a necessary preceding step for *merging*, and a *repairing* step can be included in the matching process or performed separately. Ontology *matching* identifies semantic correspondences (mainly similarities) between entities from the input ontologies, whereas ontology *merging* merges or links the corresponded entities to form the integrated ontology. Current automated ontology matching tools are becoming more and more sophisticated. They often generate a quite reliable alignment between two ontologies by finding *equivalence* relations between ontological entities, especially between concepts or instances [5]. Many ontology matching tools are publicly available such as *COMA++*^1^ [6], [7], *Falcon-AO*^2^ [8], [9], *LogMap*^3^ [10], [11], *YAM++*^4^ [12], and *AML*^5^ [13], [14], *etc*.

Despite the research interest in ontology integration and the intense activity within the community during the past two decades, the topic is still challenging as fully reliable solutions are not yet available. The practical challenge of ontology integration increases with the scale of the target system. The latter can contain numerous ontologies with hundreds of thousands of entities and axioms, becoming a pressing requirement. As a scalable context normally requires automated integration, the holistic approach, which performs a simultaneous integration of multiple ontologies in a single step, may increase the agility of the underlying methods.

In this paper, we introduce a holistic alignment-based method for integrating multiple ontologies in a customized manner; then we investigate its impact on the resulting integrated ontology. Our method takes as input two or more ontologies having overlapping domains and one or more pairwise alignment(s) between them and returns a new refactored ontology that faithfully preserves all knowledge of the input ontologies and alignments. This article is actually a *method article* that is associated with a previous publication [1]. It focuses on the methodological aspects of our holistic ontology integration approach that was briefly presented in [1]. The previously published article [1] is a *survey* that reviews the relevant literature in the ontology integration area.

*Structure of the paper* The remainder of the paper is structured as follows. Section 2 recalls some background knowledge including *ontology, OWL, ontology alignment* and *ontology refactoring* definitions, as well as ontology integration types. Section 3 describes the *ontology refactoring* process that we use in our method. Section 4 introduces our proposed ontology integration method and describes in detail its two implementations OIAR and AROM, respectively. Section 5 relies on OIAR and AROM to perform a holistic integration of multiple real-world ontologies, discusses the results, and derives the main findings. Finally, Section 6 concludes the paper with a short summary.

## 2 Preliminaries and key notions

This section provides an overview of key concepts, including *Ontology, Web Ontology Language, Ontology Alignment, Refactoring and Integration*. Additionally, we briefly analyze the different strategies for ontology integration.

### 2.1 Ontology

A largely accepted generic definition of an *ontology* is provided in [15]: “*a formal, explicit specification of a shared conceptualization (of a domain of discourse)*”. Such a concept becomes central to formally represent knowledge in a machine-processable context [2]. Additionally, ontologies are understood as rich data models to support automatic reasoning and complex query.

An ontology can be viewed as a labelled directed graph whose *nodes* are entities, and *edges* are relations. Nodes are labelled by entity names, and edges are labelled by relation names. An ontology can also be viewed as a set of triplets <$entity_{1}$, relation, $entity_{2}$>. In general, there are four types of entities [16]: *concepts* (or *classes*), *individuals* (or *instances* of classes), *object properties* (*i.e., relationships* among individuals), and *datatype properties* (*i.e., attributes* associated with individuals). An additional type of entities (*annotation properties*) is used to add human-readable metadata (such as *labels* and *comments*) at different levels of the ontology. Concepts and properties are organized within hierarchies using the built-in *subsumption*/*is-a* relation. In the abstract syntax, an ontology is a sequence of logical and non-logical axioms (rules or constraints) that express entities and their associated declarations and assertions.

### 2.2 Web ontology language

In the modern and continuously growing technological scenario, ontologies are intrinsically understood like Web Ontologies, which adopt the Web infrastructure to establish an interoperable global environment, normally referred to as Semantic Web [3]. World Wide Web Consortium (W3C)^6^ is very active in the definition of the Semantic Web standards. The most widely used languages to define ontologies are RDF (Resource Definition Framework) [17], [18], RDFS (RDF Schema) [19], and OWL (Ontology Web Language) [20], [21]. In this work, we implicitly assume OWL ontologies, since OWL endows machines with a greater ability to interpret Web content thanks to its rich vocabulary and underlying formal semantics of Description Logics. Description Logics (DL) [22] are decidable fragments of First-Order logic that are specifically designed to represent and reason on structured knowledge. Therefore, OWL ontologies are actually logical theories.

### 2.3 Ontology alignment

An alignment is the result of an ontology matching process. It is a set of semantic correspondences between two matched ontologies, denoted by $A=\{C_{1},C_{2},\;\ldots ,C_{n}\}$. Given two matched ontologies $O_{1}$ and $O_{2}$, a correspondence can be viewed as a triple $<e_{O_{1}}\;r\;e_{O_{2}}>$. More precisely, in an RDF alignment, a correspondence $C_{i}$ is a 4-tuple $<e_{O_{1}},e_{O_{2}},r,n>$ [16] such that:

• $e_{O_{1}}$ and $e_{O_{2}}$ are the members of the correspondence, where $e_{O_{1}}$ is an entity belonging to $O_{1}$, and $e_{O_{2}}$ is an entity belonging to $O_{2}$.
• r is a binary semantic relation holding or intended to hold between $e_{O_{1}}$ and $e_{O_{2}}$, such as *equivalence* (≡), *subsumption* (⊑/⊒), *disjointness* (⊥), *instantiation, etc*. In an RDF alignment, relations are flagged by one of the following symbols: ” **=**” (*i.e.* equivalent to), ”>” (*i.e.* subsumes or is more general than), ”<” (*i.e.* is subsumed by or is more specific than), and ”%” (*i.e.* incompatible with).
• n is a real number, ranging between [0,1], reflecting the confidence measure of the identified relation. It indicates the degree of trust (correctness, reliability, or truth) of the correspondence. The higher the confidence value, the more likely the relation holds [16]. In the *equivalence* case, n reflects the similarity degree.

A correspondence $C_{i}$ asserts that the relation r links $e_{O_{1}}$ and $e_{O_{2}}$ with a confidence value equal to n.

### 2.4 Ontology refactoring

Web ontologies adopt IRI/URI (Internationalized/Uniform Resource Identifier) to uniquely identify an ontology or an ontology entity. An entity IRI (or full name) assumes a *prefix* and a *suffix* as follows:



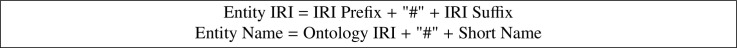



The full IRI (or the prefixed/full name) of an entity—a class, a property, or an individual—is composed of a *prefix* followed by a *suffix*. The IRI *prefix* is usually the IRI of the ontology in which the entity appears (*e.g.*, the IRI of the current ontology, or the IRI of another existing ontology). The IRI *suffix* is the short, local, or abbreviated name of the entity. The entity *prefix* and *suffix* are usually separated by a ”#” sign (they can also be separated by ”/” or ”:” signs).

The uniqueness of IRIs supports Semantic Interoperability across the Web, as the same IRI corresponds to the same semantic entity. We commonly understand ontology refactoring as a process that changes the terminology or the structure of an ontology but preserves its semantics [23]. In this work, we limit refactoring to IRIs. According to common standards and practices, depending on strategy and application, an integrated ontology may assume refactoring (original IRIs are not preserved) or not.

### 2.5 Ontology integration

Ontology integration implies the notion of *inclusion*, which refers to an enrichment/extension of an ontology by adding external knowledge into it [16], [24]. The added knowledge can be a whole ontology or a part of an ontology. Therefore, ontology *integration* also implies the notion of ontology *merging*. In fact, the integration of two ontologies is intuitively understood as merging them into a unique one. In other words, the result of including an ontology into another is equivalent to the result of merging them. Thus, ontology *merging* is a special case of ontology *integration*, and the resulting *merged* ontology can also be called an *integrated* ontology. In general, ontology *integration* is often associated with ontology *merging*, and, indeed, the two terms are often considered to be synonyms in the literature [1].

### 2.6 Ontology integration types

De Bruijn et al. [25] distinguished two ontology integration types: (*i*) the *simple merge* which is used for example, where cooperative companies look for unifying their knowledge without changing their basic ontologies and data associated with them; and (*ii*) the *full merge* which is used, for example, in cases where two newly merged companies look for completely unifying their knowledge. Both types of merging are thoroughly described in the following.

#### 2.6.1 Simple merge (bridge ontology)

**Fig. 1:**
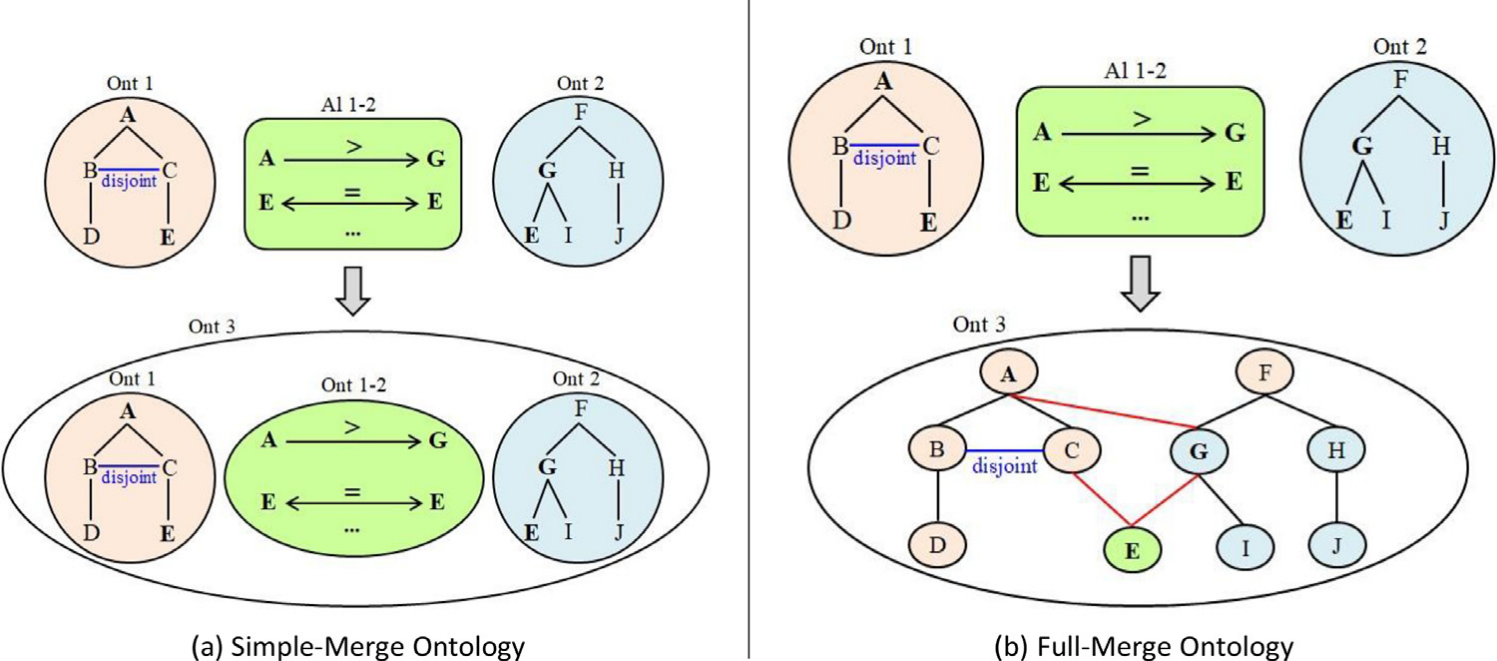
Ontology integration types.

The *Simple Merge* (*a.k.a* the *Simple Union* [26]) imports the input ontologies into a new one—constituting a union of input ontologies—and adds bridging axioms, called *articulations*, translating the alignment between them (*See* Fig. 1a). These added axioms are actually semantic correspondences interpreted as or transformed into ontological statements to bridge the overlapping part of the input ontologies. In this type of integration, equivalent entities in the integrated ontology are mentioned more than once but considered as non-redundant since they are linked by *equivalence* axioms (*See* Fig. 1a). The W3C best practices group [27] recommends integrating ontologies in the OWL language and interpreting correspondences between them as OWL axioms. The *subsumption* correspondences, between classes and properties, are expressed by built-in *subClassOf* and *subPropertyOf* OWL axioms, respectively. The *equivalence* correspondences between classes, properties and individuals are expressed by built-in *equivalentClass, equivalentProperty* and *sameAs* OWL axioms, respectively. The *disjointness* correspondences between classes, properties and individuals are expressed by built-in *disjointWith, propertyDisjointWith* and *differentFrom* OWL axioms, respectively. Therefore, the correspondences of the alignment A can be perceived as an ontology $O_{A}$ called *articulation ontology* [28], *intersection ontology* [29], or *intermediate ontology* [30]. In the case of two input ontologies, the integrated ontology $O_{3}$ is viewed as the union of $O_{1}$, $O_{2}$ and $O_{A}$ where $O_{3}=O_{1}\cup O_{2}\cup O_{A}$ [31]. The resulting ontology is generally called a *bridge ontology* (*a.k.a* a *merged* ontology or an *integrated* ontology).

To achieve ontology modularization, the OWL ontology language provides a built-in import statement <*owl:imports*>. The import statement includes the content of an entire ontology into the current ontology by only referencing the URI or the local file of that ontology. Therefore, most of the state-of-the-art approaches get the integrated ontology $O_{3}$ by creating an empty ontology that directly imports $O_{1}$, $O_{2}$ and $O_{A}$, after converting the RDF alignment A to an OWL ontology $O_{A}$. Otherwise, the integrated ontology $O_{3}$ is obtained by importing the two input ontologies $O_{1}$ and $O_{2}$ into $O_{A}$; so that $O_{A}$ becomes $O_{3}$. The import is automatically performed by simply declaring the OWL import statements referencing the ontologies to be imported. This solution clearly favors modularity and reusability in the Semantic Web; However, it is not a generic solution since we cannot customize the imported ontologies in the integrated ontology. In some ontology development tasks, ontology developers may need to customize the imported ontologies, *e.g.*, by importing only a part of them or by refactoring their IRIs/namespaces and some of their entity names *etc*. Thus, this solution is not particularly suitable for the area of ontology development, despite its huge advantage for the Semantic Web. From all the inspected state-of-the-art ontology integration works, the only work that includes refactoring in its integration process is the paper of Ziemba et al. [32]. However, the major disadvantage of this work is being completely manual and thus impossible to apply for large ontologies.

#### 2.6.2 Full merge

The *Full Merge* [26] (*a.k.a.* the *Complete Merge* [25], [33]) imports the input ontologies into a new ontology—constituting a union of input ontologies—and merges each set of equivalent entities into a single new entity that preserves all their attached description and relations (*See* Fig. 1b). The resulting merged entities will be represented only once in the merged ontology, which avoids the existence of redundant entities. However, the multiple inheritance does exist in the merged ontology, since each merged entity is assigned to more than one direct parent, where each parent comes from an input ontology (*See* Fig. 1b). Ontological axioms, constituting the merged ontology and originating from the input ontologies, are updated by replacing every occurrence of the original entities with its newly merged entity. That is each axiom, in which appears the name of one of the entities that have been merged, must be updated by replacing the name of that original entity with the name of the newly merged one. In the literature, authors identify the merged entities by either a unique (alphanumeric) code or by the name of one of the original entities that have been merged—commonly, the name of the entity that belongs to the preferred input ontology; then, they add the short names of the original entities (that have been merged) as additional labels to the newly merged entity. *Subsumption* axioms can be added to link subsuming and subsumed entities, as prescribed in the alignment(s). With two input ontologies, the merged ontology $O_{3}$ can be viewed as the union of $O_{1}$ and $O_{2}$ where $O_{3}=O_{1}\cup O_{2}=\left(O_{1}-O_{2}\right)\cup \left(O_{2}-O_{1}\right)\cup \left(O_{1}\cap O_{2}\right)$ [25]. The resulting ontology can be referred to as a *unified* ontology, a *merged* or an *integrated* ontology.

Many research works are not generic in terms of the number of input ontologies to integrate: They are tailored to integrate only two ontologies because the process of matching and integrating more than two ontologies at the same time is much more complex, *e.g*, in [24], [31], [34], [35], [36], [37], [38], [39], [40], [41], [42], [43], [44], [45], [46], [47]. In order to integrate multiple ontologies, these works had to perform an iterative incremental process that implements a series of pairwise ontology matching and integration, *e.g.*, the works of [48], [32] and [24], *etc*.

In the remainder of this paper, we introduce a generic ontology integration method. It integrates two or more input ontologies in a non-incremental (*i.e.*, holistic) manner, using pairwise alignments. It effectively includes the input ontologies in the integrated ontology and refactors the names of all the included entities, if requested.

## 3 Ontology refactoring in our method

We have made two versions of our method implementation: (*i*) a non-refactored version, and (*ii*) a refactored version. The ontology refactoring aims to customize our resulting integrated ontology.

In an ontology, we cannot have two identical IRIs for two entities of the same type, because it will be considered as the same entity. According to the standards, we would like that all entities (of our output integrated ontology) to have our new ontology’s IRI as a prefix. However, when we integrate ontologies from the same domain, different entities (belonging to different ontologies) can have the same short name. For example, ”*Conference*”, ”*Paper*”, ”*Author*” and many other classes exist in at least three ontologies from the *Conference*^7^ OAEI track, namely the ontologies *cmt* ($O_{1}$), *conference* ($O_{2}$), and *confOf* ($O_{3}$). Here are the original full names/IRIs of the ”*Conference*” classes belonging to each ontology:

http://cmt#Conference

http://conference#Conference

http://confOf#Conference

They are semantically equivalent (≡) classes expressed in the three pairwise alignments between *cmt, conference* and *confOf* ontologies as follows:

http://cmt#Conference ≡ http://conference#Conference

http://cmt#Conference ≡ http://confOf#Conference

http://conference#Conference ≡ http://confOf#Conference

These classes will correctly appear in the integrated ontology resulting from a non-refactored simple merge. However, in the refactored version, if the IRI of our output ontology is, for example, ”*http://integration*”, then these classes will not appear as shown below:

http://integration#Conference

http://integration#Conference

http://integration#Conference

That is impossible, since a full IRI of a class cannot be assigned to more than one class. To overcome this problem, we have chosen to add an ID to the IRI prefix of all the entities.



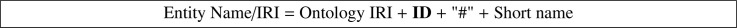



To do so, we assign a number to each input ontology. The first parsed ontology will have the number 1, the second parsed ontology will have the number 2, and so on. The ID represents the number of the ontology from which an entity originates. We have set the ID to four characters, so the last four characters of the IRI prefix of each entity will be reserved for the ID. That is, if the ontology number (N) is less than 10 (*i.e.*, in case we have integrated less than 10 ontologies at the same time), then the ID will be ”/00N”; if the ontology number (NN) is greater than 10 and less than 100 (*i.e.*, in case we have integrated more than 10 ontologies at the same time), then the ID will be ”/0NN”; and if the ontology number (NNN) is greater than 99 (*i.e.*, in case we have integrated more than 99 ontologies at the same time), then the ID will be ”/NNN”. This is how the full IRIs of the ”*Conference*” classes will appear in the integrated ontology resulting from a refactored simple merge:

http://integration **/001** #Conference

http://integration **/002** #Conference

http://integration **/003** #Conference

Doing so, all entities will have a unique customized IRI in the integrated ontology, and all their attached description will be preserved correctly. Besides, this is how we can differentiate entities and directly track back their origin (*i.e.*, discover from which ontology they are derived). In the non-refactored simple merge, if the input ontologies contain some common entities that have the same full IRI (which generally refers to an already existing entity from another ontology), then these entities will be automatically merged and stated only once in our integrated ontology—like what would happen for ”*http://integration#Conference*” classes in the last example. In the full-merge ontology, we have assigned an ID “èè/000” to the merged entities (resulting from the merge of the sets of equivalent entities). So, this is how the full IRI of the merged class ”*Conference*” will appear in the merged ontology resulting from a refactored full merge:

http://integration **/000** #Conference

In the refactored version of our implementation, the output integrated ontology will rather be perceived as a new original ontology, as if it was not the result of an integration, since all of its entities have an IRI prefix specific to us.

## 4 Method details

**Fig. 2:**
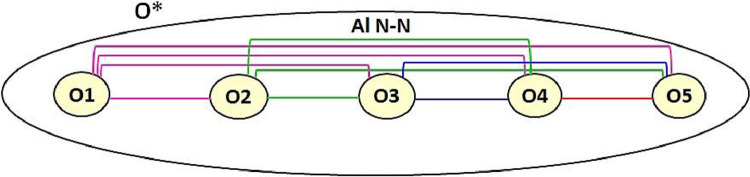
Holistic Ontology Integration using Pairwise Alignments.

In this paper, we propose a holistic ontology integration method that effectively integrates several input ontologies in a unique one and refactors entity names accordingly if requested. It integrates multiple ontologies using pairwise alignments between all pairs of ontologies, as shown in Fig. 2. *Holistic* or N*-ary* ontology integration combines all the input (or *source*) ontologies $O_{1}$, $O_{2}$,..., $O_{n}$ in a single iteration, *i.e.* in a non-incremental manner, to constitute the output (or *target*) integrated ontology $O^{*}$. It is a scalable approach since it is suitable for a large number of input ontologies. We should note that we do not perform any ontology matching process; Our method takes external alignments as input. Indeed, we rather leverage the advances made in the ontology matching area by using external alignments such as alignments generated by top-performing matching tools (*e.g., LogMap*) or reference alignments. Using these reliable pairwise alignments will help us integrate large and complex ontologies even without having a robust matching tool.

In the following subsections, we thoroughly describe *OIAR* (Ontology Integration with Alignment Reuse) and *AROM* (Alignment Reuse for Ontology Merging), the two proposed algorithms that implement holistic ontology integration. Both implementations support refactored as well as non-refactored integration, while they provide a different kind of integration. OIAR targets *simple merge* integration as previously defined, and AROM aims to *full merge* integration. They both take as input two or more OWL ontologies to be integrated, one or more RDF alignments between them (written in the Alignment API format^8^ [49]), a new URI or IRI as a namespace for the output integrated ontology, and a confidence threshold ranging between [0,1] to trim correspondences of the input alignment(s). The user selects the input ontologies and alignments; and input ontologies should cover overlapping or complementary domains. We report a summary of the most characterizing features for each algorithm in Table 1.

**Table 1:** Method overview.

Algorithm	Method	Integration type	Alignment	Refactored	Non-refactored
*OIAR*	Holistic Integration	Simple Merge	External	✓	✓
*AROM*	Holistic Integration	Full Merge	External	✓	✓

### 4.1 OIAR

**Fig. 3:**
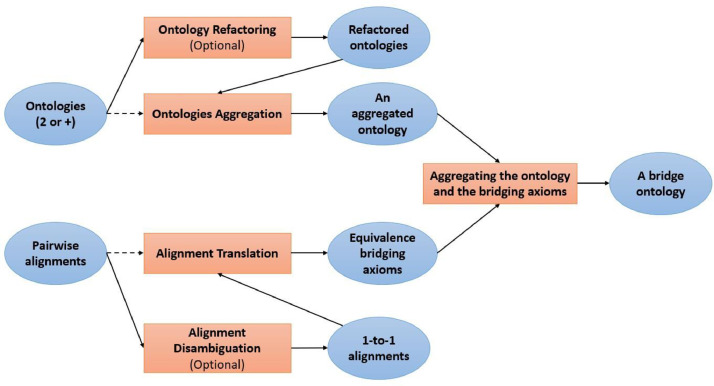
OIAR General steps.

In this subsection, we introduce the *Ontology Integration with Alignments Reuse* (OIAR) algorithm. The latter aims to automatically build a bridge ontology among several input ontologies. Fig. 3 shows the general steps of the OIAR process. The current OIAR framework includes two different versions which provide respectively a non-refactored output (based on original IRIs) and a refactored output (based on modified IRIs). Both versions take into input the ontologies (in OWL) to be integrated, as well as the alignments (in RDF) among them. OIAR source code and other associated resources are freely available on GitHub^9^. We describe the details of the two versions of the method in the following subsections.

#### 4.1.1 OIAR General process – refactored version

The refactored version of the OIAR algorithm comprises the following steps:

1. Loading two or more input ontologies (owl files) and alignment(s) between them (rdf file(s));
2. Parsing all the axioms of the input ontologies and creating exactly the same refactored axioms corresponding to them;
3. Disambiguating the input alignments by transforming them from 1-to-N to 1-to-1 alignments (optional);
4. Parsing the correspondences of the input alignments and creating refactored *bridging* axioms corresponding to them;
5. Creating the output integrated ontology (*i.e.*, an owl file) by summing all the created OWL axioms (axioms of steps 2 and 4).

In the next paragraphs, we thoroughly describe OIAR steps separately.

***Step 1**: Loading Input Ontologies & Alignments*

After selecting and entering the ontologies to be integrated and the alignments between them, the input ontologies are loaded in the OWL API Manager^10^. The latter is the central point of access in OWL API [50] since it is used to load, create, and access ontologies.

***Step 2**: Creating Refactored Copies of Axioms of the Input Ontologies*

First, we create four HashMaps:

1. The *classes* HashMap, whose ”key” contains the original IRI of a given class, and ”value” contains its associated ID (which is the number of the ontology from which that class originates);
2. The *object properties*’ HashMap, whose ”key” contains the original IRI of a given object property, and ”value” contains its associated ID (which is the number of the ontology from which that property originates);
3. The *data properties*’ HashMap, whose ”key” contains the original IRI of a given data property, and ”value” contains its associated ID (which is the number of the ontology from which that property originates); and finally
4. The *individuals* HashMap, whose ”key” contains the original IRI of a class instance, and ”value” contains its associated ID (which is the number of the ontology from which that individual originates).

• While parsing the classes of the input ontologies, we fill up the first HashMap (for future use) and we extract, for each parsed class, its name and definition/description (*i.e.*, its *super, equivalent* and *disjoint* class expressions, and its annotations–*labels, comments*, and *annotation properties*–, *etc*), information with which we create a refactored copy of that class and its definition in our output integrated ontology. In fact, for each parsed class, we replace its original IRI prefix and those of all entities mentioned in its definition by the IRI of our output ontology + the number (ID) of the currently parsed ontology.

• While parsing the object properties and data properties of the input ontologies, we fill up the second and third HashMaps (for future use), and we extract, for each parsed property, its name and definition (*i.e.*, its *domains* and *ranges* class expressions (or *data ranges*), its *super, inverse, equivalent* and *disjoint* property expressions, its characteristics, and its annotations–*labels, comments*, and *annotation properties*–, *etc*), information with which we create a refactored copy of that property in our output integrated ontology. In fact, for each parsed property, we replace its original IRI prefix and those of all entities mentioned in its definition by the IRI of our output ontology + the number (ID) of the currently parsed ontology.

• While parsing the individuals/instances of the input ontologies, we fill up the fourth HashMap (for future use), and we extract, for each parsed individual, its name and definition (*i.e.*, its *class assertions*, its negative and positive *property assertions*, its *sameAs* and *different* individuals, and its annotations–*labels, comments*, and *annotation properties*–, *etc*), information with which we create a refactored copy of that individual in our output integrated ontology. In fact, for each parsed individual, we replace its original IRI prefix and those of all entities mentioned in its definition by the IRI of our output ontology + the number (ID) of the currently parsed ontology.

***Step 3**: Disambiguating the Input Alignments (Optional)*

An ambiguous alignment [16] allows to match the same entity from a first ontology to several entities from a second ontology. In other words, it contains some correspondences that share an entity in common: either a *source* entity (*i.e.*, from $O_{1}$), or a *target* entity (*i.e.*, from $O_{2}$). These correspondences are called *ambiguous correspondences* [16], *correspondences of higher-multiplicity* [24] or *higher-multiplicity correspondences* [24]. That is, entities composing an ambiguous correspondence are involved in other correspondences, such that a *source* entity or a *target* entity occurs in at least two correspondences. The following example shows three ambiguous correspondences:

$O_{1}$ :Student ≡ $O_{2}$ :Student

$O_{1}$ :Student ≡ $O_{2}$ :Scholar

$O_{1}$ :Student ≡ $O_{2}$ :PhD_Student

In general, alignments between independently developed ontologies are *many-to-many* alignments (of cardinalities n:m or *:*), where zero or more entities from the first ontology can match with zero or more entities from the second ontology. Therefore, *many-to-many* alignments are actually ambiguous alignments. Whereas, a *one-to-one* alignment (of cardinalities 1:1) can only match an entity from a first ontology to a single entity from a second ontology; so *source* entities (from $O_{1}$) and *target* entities (from $O_{2}$) appear in at most one correspondence [51].

Alignment disambiguation aims to convert a *many-to-many* alignment to a *one-to-one* alignment. To do so, OIAR filters correspondences having the same *source* entity or the same *target* entity by only keeping the most confident correspondence (having the highest confidence value) and removing the remaining ones. This approach is based on the intuition assuming that among the ambiguous *equivalence* correspondences, there is a single correct correspondence that reflects a true synonym, while the remaining ones are rather similar, related, or overlapping terms [24].

**Fig. 4:**
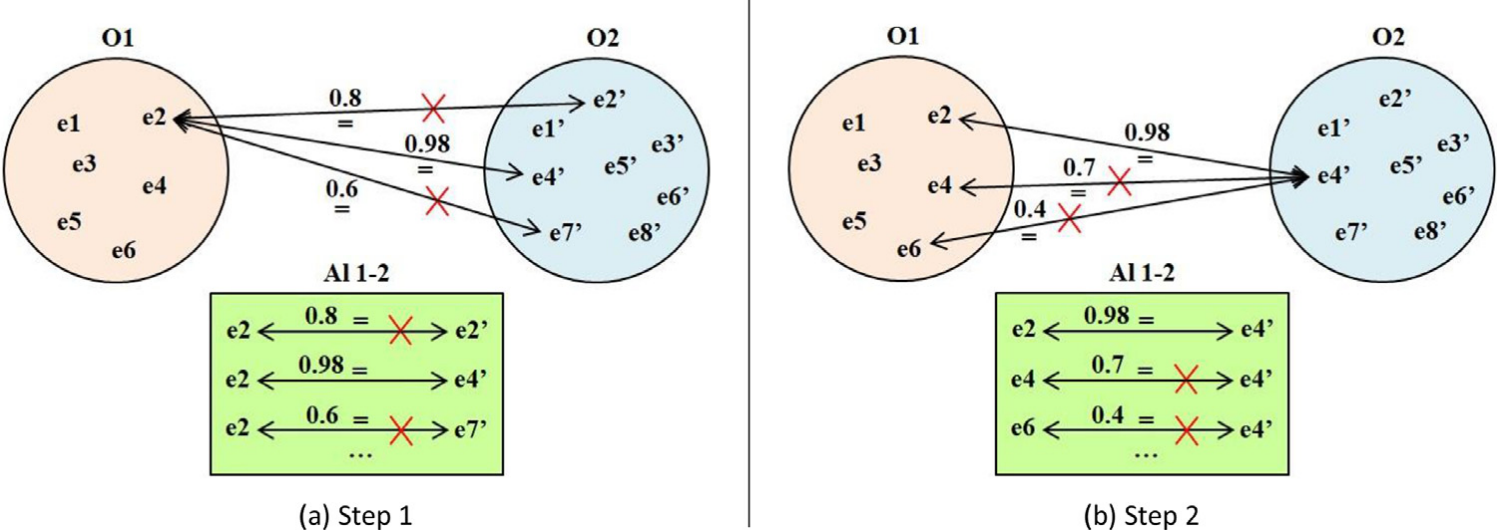
Alignment disambiguation process.

OIAR first filters out correspondences having the same *source* entity (as shown in Fig. 4a); then, it filters out correspondences having the same *target* entity (as shown in Fig. 4b) by only keeping one correspondence having the highest similarity value (*See* Algorithm 1). If all ambiguous correspondences have the same confidence value, then OIAR keeps all of them because if it randomly chooses one of them, then results will differ for each chosen correspondence (*See* Algorithm 1). OIAR involves this algorithm to disambiguate not only ambiguous *equivalence* correspondences, but also ambiguous *subsumption* and *disjointness* correspondences.

***Step 4**: Creating Refactored Bridging Axioms Translating the Input Alignments* In practice, we cannot link different types of entities by the same axioms. In OWL API [50], there are four types of methods for creating *bridging* axioms; each one is dedicated to a particular type of entities (classes, object properties, data properties, and individuals). For example, to create an *equivalence* axiom between two classes class1 and class2, we should call the following OWL API method: datafactory.getOWLEquivalentClassesAxiom(class1, class2). The same goes for creating *equivalence* axioms between two object properties, two data properties, or two individuals. However, in an RDF alignment, it is impossible to directly identify the type of the matched entities because they are only expressed by their original full IRIs—as they were defined in their original ontology. We cannot identify whether a given entity IRI represents a class, a property, or an individual. For this reason, we use the four HashMaps already filled in the second step (Section 4.1.1) to directly identify the type and the ID of each pair of entities in a correspondence. By doing so, we will be able to create *bridging* axioms for pairs of classes, object properties, data properties, or individuals.

Trimming an alignment consists of removing correspondences that have a confidence value below a given threshold, in order to ensure that only the most confident correspondences are kept. Trimming applies an α-cut to the alignment, such that the confidence threshold α∈[0,1]. After choosing a given threshold as input, OIAR automatically trims the input alignments using the predefined method cut() of the Alignment API [49].

While parsing the correspondences of the input alignments after being trimmed, we create refactored *bridging* axioms (semantic links) that exactly translate the parsed correspondences, and add them to our output ontology. The refactoring is made by replacing the original IRI prefixes of the two entities of each correspondence with the IRI of our output ontology + the associated ID (the number assigned to the original ontology). The created *bridging* axioms can be *equivalence, subsumption*, or *disjointness* axioms, according to the relation type of the parsed correspondence. At last, the *bridging* axioms of our output integrated ontology will rather be perceived as normal axioms linking entities of a new original ontology, as if it was not the result of an integration, since all of its entities have a refactored IRI prefix.

***Step 5**: Creating the Output Integrated Ontology*

**Fig. 5:**
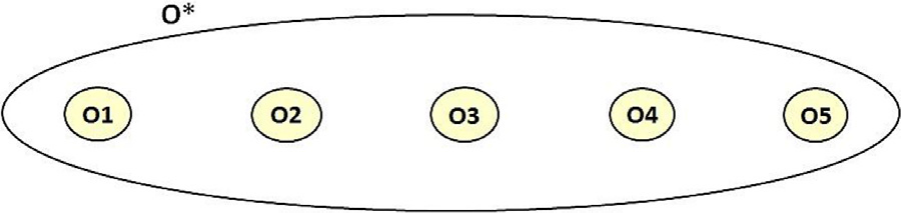
Ontology aggregation/composition.

When we execute all steps except step 4, we obtain an *aggregated* ontology, as shown in Fig. 5; and when we execute all steps, we obtain an *integrated* ontology—generally called a *bridge ontology*, as shown in Fig. 1a. Indeed, axioms of step 2 will form an OWL ontology that simply aggregates the input ontologies without making any semantic interoperability between them. However, axioms of step 4 are *bridging* axioms that will form, together with the axioms of step 2, an OWL bridge ontology that allows the aggregated ontologies to semantically interoperate via the *bridging* axioms. We will finally get an owl file corresponding to the output ontology.

#### 4.1.2 OIAR General process – non-refactored version

The non-refactored version of the method differs from the previously described one, as it keeps the original IRIs of the entities from the different input ontologies. The algorithm of the non-refactored version of OIAR is structurally similar to the refactored one and can be summarized in the following steps:

1. Loading two or more input ontologies (owl files), and alignment(s) between them (rdf file(s));
2. Automatically aggregating all the axioms of the input ontologies—using the OWLOntologyMerger() method of OWL API;
3. Disambiguating the input alignments by transforming them from 1-to-N to 1-to-1 alignments (*See* Section 4.1.1) (optional);
4. Translating the correspondences of the input alignments into OWL *bridging* axioms;
5. Creating the owl file of the output integrated ontology by summing all the axioms (axioms of steps 2 and 4).

This approach consists of the automatic aggregation of the input ontologies and the ontologies corresponding to the input alignments. Suppose that we have two input ontologies $O_{1}$ and $O_{2}$ to be integrated, and an input alignment A between them. This approach first aggregates $O_{1}$ and $O_{2}$ to get the ontology $O_{12}$, then aggregates $O_{12}$ and $O_{A}$ to get the output integrated ontology $O_{3}$, where $O_{A}$ is an ontology generated by converting A into a set of OWL axioms. The approach can be considered as aggregating $O_{1}$, $O_{2}$ and $O_{A}$ (*i.e.*, $O_{3}=O_{1}+O_{2}+O_{A}$) without modifying neither the input ontologies nor the input alignments. In the next subsections, we will detail the main steps of this OIAR version, namely steps 2 and 4.

***Step 2**: Aggregating the Axioms of the Input Ontologies*

Aggregating ontologies using the OWL API is straightforward. First, we use the predefined method OWLOntologyMerger(), which automatically aggregates all the ontologies that were loaded into the OWLOntologyManager. Then, we just need to specify an ontology IRI to the predefined method createMergedOntology(), which will return an aggregated OWL ontology having that specified IRI as namespace. The returned aggregated ontology does not miss any knowledge from the input ontologies and does not alter any axiom.

It is worth mentioning that the terms *OntologyMerger* and *MergedOntology*, used as names for the OWL API methods, further stress the confusion associated with the term *merging* in the community. Actually, these OWL API methods do not perform an ontology merge, but rather a simple aggregation/composition/concatenation of the input ontologies (*See* Fig. 5). Moreover, the Protégé [52] ontology editor makes exactly the same mistake with the option ”*Merge ontologies*” of its ”*refactor*” menu.

***Step 4**: Creating Bridging Axioms Translating the Input Alignments*

The Alignment format, *a.k.a.* the *RDF Alignment format* or the *Alignment API format*, is expressed in the RDF (Resource Description Framework) language. It is a freely extensible format, therefore, any alignment A expressed by this format can be automatically transformed into OWL bridging axioms making up an ”intermediate” ontology $O_{A}$. The OWLAxiomsRendererVisitor() method of the Alignment API automatically transforms the alignment correspondences into *equivalence, subsumption* and *disjointness* bridging axioms. Unfortunately, we could not complete the alignment transformation task in this way. Therefore, instead of using the Alignment API in step 4, we applied the same idea of the refactored version of OIAR (*See* Section 4.1.1). It consists in parsing the correspondences of the input alignments and creating their associated OWL *bridging* axioms, as follows:

• While parsing the classes of the input ontologies, we fill the *classes* HashSet with the original IRIs of the parsed classes.
• While parsing the object properties of the input ontologies, we fill the *object properties*’ HashSet with the original IRIs of the parsed object properties.
• While parsing the data properties of the input ontologies, we fill the *data properties*’ HashSet by the original IRIs of the parsed data properties.
• While parsing the individuals of the input ontologies, we fill the *individuals* HashSet with the original IRIs of the parsed individuals.

Remember that in the OWL API, *bridging* axioms between entity pairs can only be created by using specific methods dedicated for each type of entities; Besides, in an alignment, it is impossible to know the type of the matched entities since they are only expressed by their original IRIs. For this reason, we use the four already filled HashSets to directly identify the type of each entity pair composing correspondences of an alignment. By doing so, we will be able to create *bridging* axioms for all types of entities.

While parsing the correspondences of the input alignments after being trimmed, we create *bridging* axioms that exactly translate the parsed correspondences—without altering any original IRI—and we add them to the initial aggregated ontology generated by the previous step.

### 4.2 AROM

**Fig. 6:**
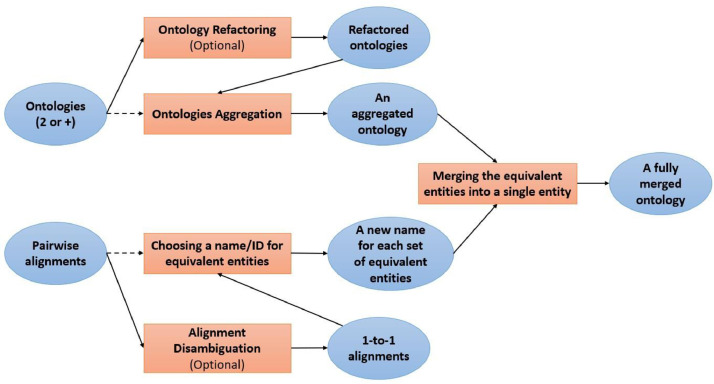
AROM General steps.

In this subsection, we introduce the *Alignments Reuse for Ontology Merging* (AROM) algorithm. The latter aims to automatically build a full-merge ontology among several input ontologies. Fig. 6 shows the general steps of the AROM process. The current AROM framework includes two different versions which provide respectively a non-refactored output (based on original IRIs) and a refactored output (based on modified IRIs). Both versions take into input the ontologies (in OWL) to be integrated, as well as the alignments (in RDF) among them. AROM source code and other associated resources are freely available on GitHub^11^.

The AROM algorithm comprises the following steps:

1. Loading two or more input ontologies (owl files) and alignment(s) between them (rdf file(s));
2. Disambiguating the input alignments by transforming them from 1-to-N to 1-to-1 alignments (*See* Section 4.1.1) (optional);
3. Merging the names of each set of equivalent entities: Parsing the *equivalence* correspondences of the input alignments, and generating a HashMap whose ”key” contains the entity to be merged, and whose ”value” contains the merged new name of that entity;
4. Parsing all the axioms of the input ontologies, and creating exactly the same (refactored) axioms corresponding to them, such that each entity appearing in the axioms is replaced by its new merged name (whenever it is mentioned in the input alignments, therefore whenever it exists as a key in the HashMap);
5. If there are any *subsumption* (⊑, ⊒) or *disjointness* (⊥) correspondences in the input alignments, then creating (refactored) *bridging* axioms corresponding to them, such that each entity appearing in these axioms is replaced by its merged new name (whenever it exists as a key in the HashMap);
6. Creating the owl file of the output merged ontology by summing all the created OWL axioms (*i.e.*, axioms of steps 4 and 5).

Remark 1 If the input alignments only contain *equivalence* correspondences (*i.e.*, they do not contain any *subsumption* nor *disjointness* correspondence), then it is useless to execute step 5.

In the following paragraphs, we thoroughly describe each step separately.

***Step 1**: Loading Input Ontologies & Alignments*

This step is the same step used in OIAR (*See* Section 4.1.1).

***Step 2**: Disambiguating the Input Alignments (Optional)*

This step is the same step used in OIAR (*See* Section 4.1.1).

***Step 3**: Generating a New Name for each Set of Equivalent Entities to be Merged*

Algorithm 2 parses the *equivalence* correspondences of the input alignments after being trimmed, and returns a HashMap whose ”key” contains the original IRI of a given entity, and ”value” contains its new name (or code) in our future merged ontology. By doing so, we associate a unique code to each set of equivalent entities. We will use this unique code as a short name for the merged entity resulting from the merge of a set of equivalent entities in the merged ontology. The resulting merged entity will also have new labels that are actually the short original names of the entities that were merged into it.

**Algorithm 2:**
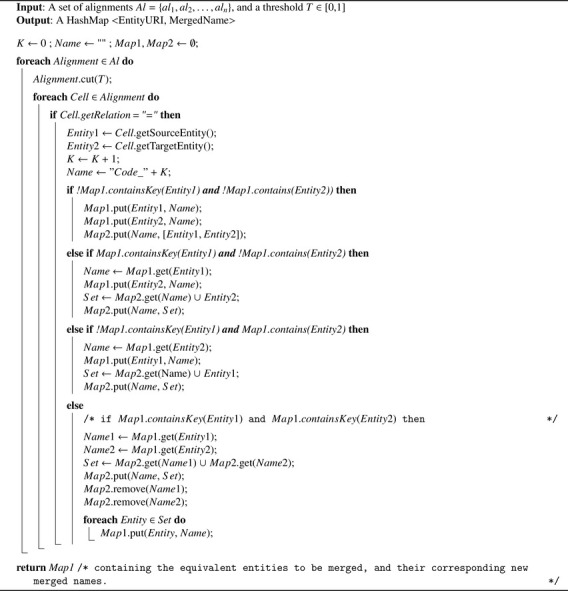
Merging Equivalent Entities’ Names.

***Step 4**: Creating (Refactored) Merged Entities (or Merging Equivalent Entities)*

• We parse the classes of the input ontologies, and we extract their IRIs and descriptions (*i.e.*, their *super, equivalent* and *disjoint* class expressions, and their annotations—*labels, comments* and *annotation properties*—, *etc*). If a parsed class or one of the entities in its description exists as a ”key” in the HashMap (resulting from the previous step), then we replace it by its ”value” (*i.e.*, by its new merged name). In other words, in our merged ontology, we create a (refactored) copy of that class and its description, such that each entity occurrence is replaced by its associated new name. If the parsed class is a merged one, then we also add its original short name as a label in its new description.
• We parse the object properties and data properties of the input ontologies, and we extract their IRIs and descriptions (*i.e.*, their *super, inverse, equivalent* and *disjoint* property expressions, their *domains* and *ranges* class expressions (or *data ranges*), their characteristics, and their annotations—*labels, comments*, and *annotation properties*—, *etc*). If a parsed property or one of the entities in its description does exist as a ”key” in the HashMap, then we replace it by its ”value” (*i.e.*, by its new merged name). In other words, in our merged ontology, we create a (refactored) copy of that property and its description, such that each entity occurrence is replaced by its associated new name. If the parsed property is a merged one, then we also add its original short name as a label in its new description.
• We parse the individuals/instances of the input ontologies, and we extract their IRIs and descriptions (*i.e.*, their *class assertions*, their negative and positive *property assertions*, their *sameAs* and *different* individuals, and their annotations—*labels, comments*, and *annotation properties*—, *etc*). If a parsed individual or one of the entities in its description exists as a ”key” in the HashMap, then we replace it by its ”value” (*i.e.*, by its new merged name). In other words, in our merged ontology, we create a (refactored) copy of that individual and its description such that each entity occurrence is replaced by its associated new name. If it is a merged individual, then we also add its original short name as a label in its new description.

***Step 5**: Creating (Refactored) Bridging Axioms Translating the Input Alignments* We parse the *subsumption* and *disjointness* correspondences of the input alignments after being trimmed. If one of the entities (of the parsed entity pairs) exists as a ”key” in the HashMap, then we replace it by its ”value” (*i.e.*, by its newly merged name). Then, we create *bridging subsumption* or *disjointness* axioms that exactly translate the parsed (refactored) correspondences, and add them to our output merged ontology.

***Step 6**: Creating the Output Merged Ontology* When we execute all steps except steps 3 and 5, we obtain an *aggregated* ontology that will simply compose/concatenate/associate the input ontologies without making any semantic interoperability between them, as shown in Fig. 5; and when we execute all steps, we obtain an *integrated* ontology—generally called a *fully merged ontology* or a *full-merge ontology*, as shown in Fig. 1b. Indeed, if and only if step 3 is executed, then axioms of the step 4 will form an OWL aggregated ontology where equivalent entities are fully merged into merged entities. After that, step 5 will add *subsumption* and *disjointness bridging* axioms (if there are any *subsumption* and *disjointness* correspondences in the input alignments). We will finally get an owl file corresponding to the merged output ontology.

## 5 Experimentation

In this section, we provide an in-depth presentation and analysis of the experiments conducted.

### 5.1 Ontology integration evaluation

On the one hand, it is difficult to make a comparison between ontology integration approaches because there are no agreed quality measures/metrics for assessing them, such as *Precision* and *Recall* for assessing ontology alignments. To the best of our knowledge, there are no references or benchmarks or gold standard metrics within the ontology integration community to objectively evaluate the quality of integration methods. Besides, it is impossible to manually obtain an ideal integration result for large ontologies, and there could be more than just one ideal result [26]. On the other hand, the related work approaches use different input ontologies, different ontology integration types, different input parameters and different evaluation metrics [1]. Therefore, it is impossible to compare our obtained results with other ontology integration methods. In conclusion, assessing ontology integration approaches is still an open issue.

It should be noted that we are not going to assess the matching results (*i.e.*, the quality of the alignments) since we will be using the OAEI reference alignments which are considered as the best possible alignments. In this work, we aim to assess the quality of the resulting integrated ontology. To do so, we will be using the following measures [1]:

1. *Entities completeness/coverage*: Number of preserved entities from the input ontologies;
2. *Axioms completeness/coverage*: Number of preserved axioms from the input ontologies;
3. *Correspondences completeness*: Number of preserved correspondences from the input alignment(s);
4. *Ontology consistency*: Is the integrated ontology consistent? (True or False);
5. *Ontology coherence*: Number of unsatisfiable classes in the integrated ontology;
6. *Entities redundancy*: Number of duplicated/redundant entities in the integrated ontology.

The three first measures assess the degree of *information preservation* or *completeness* to ensure that there is no information loss from the input ontologies and alignments. The two first metrics reflect the knowledge preservation from the input ontologies, while the third metric reflects the knowledge preservation from the input alignments. The metric of *entities coverage* measures the number (or the percentage) of preserved entities in the integrated ontology compared to an expected number of entities:

• For the *simple merge* case, the number of entities of the integrated ontology should be ideally equal to the sum of the entities of the input ontologies.
• For the *full merge* case, the number of entities of the integrated ontology cannot be easily determined. However, in the case of two ontologies, the number of entities of the integrated ontology should be ideally equal to the sum of entities of the two input ontologies, minus the number of merged entities (*i.e.*, minus the number of *equivalence* correspondences of the 1-to-1 alignment).

The metric of *axioms coverage* [24] measures the number (or the percentage) of preserved axioms in the integrated ontology. Entities and axioms of the input ontologies should ideally be completely preserved. The metric of *correspondences coverage* [45] reflects the number (or the percentage) of preserved correspondences in the integrated ontology.

The fourth and fifth metrics reflect the *consistency* and the *coherence* of the integrated ontology. The *consistency* metric evaluates the logical/semantic consistency of the integrated ontology, while the *coherence* metric measures the number (or the percentage) of unsatisfiable classes in the integrated ontology. An unsatisfiable class [53], *a.k.a.* a *coherence violation*, is a class containing a contradiction in its description, thus no individual/instance can meet all the requirements to be a member of that class. Unsatisfiable classes are called *coherence violations* because they cause the incoherence of the integrated ontology. Indeed, if there is at least one unsatisfiable entity in an ontology, then the latter becomes incoherent. Similarly, if an unsatisfiable class is instantiated (*i.e.*, have individuals as instances/members), then the integrated ontology becomes inconsistent. An inconsistent ontology [54] is an ontology that has no satisfying interpretation. Ontology inconsistency is a fatal error because we cannot infer any useful knowledge from the ontology by ontology reasoning. Overall, ontology *inconsistency* and *incoherence* are logical errors reflecting semantic conflicts/contradictions between distinct classes in the integrated ontology.

The metric of *entities redundancy* measures the number (or the percentage) of redundant entities in the integrated ontology. Redundant entities are distinct but equivalent entities, having the same meaning and representing the same entity in the integrated ontology. These entities become redundant because they are neither merged with each other, nor linked by *equivalence* axioms in the integrated ontology. Redundant entities complicate text annotation tasks due to ambiguity, increase the size of the integrated ontology, and decrease the interoperability between applications that use these entities [24].

We will also be using the following performance evaluation criteria:

1. *Runtime*: The execution time performance;
2. *Scalability*: Are runtimes scalable when using heavyweight input ontologies?;
3. *Human Intervention*: Is the user involved in the ontology integration process?

The ontology integration algorithm should have a competitive runtime compared to runtimes of the related work algorithms. In addition, it should still have an acceptable runtime and a good result, even for large and rich ontologies. Finally, the intervention of the user or the expert should be minimal; It is better to have a fully automatic algorithm without any manual effort.

### 5.2 Experimental environment

We performed all tests on a standard laptop with 4 Gb of RAM. We have implemented OIAR and AROM in Java, and we have used the following external tools:

• OWL API^12^ [50], [55] (Version 4.1.4), a Java programming interface for developing, manipulating, and serializing OWL ontologies.
• Alignment API^13^ [49], [56] (Version 4.9), a Java programming interface for expressing, accessing, and manipulating ontology alignments in the Alignment format.
• HermiT^14^ [57], [58] (Version 1.3.8), a DL reasoner for inferring implicit knowledge, interrogating and classifying ontologies, and verifying the consistency and coherence of ontologies.
• ELK^15^ [59], an EL reasoner dedicated for efficiently reasoning on large ontologies. ${\mathrm{EL}}^{++}$ [60], [61] is a fragment and a lightweight version of DL. Since HermiT cannot scale when reasoning over large OWL ontologies, we use ELK instead.

HermiT or ELK ontology reasoners are used to check the consistency of the resulting integrated ontology and to compute the number of its unsatisfiable classes.

### 5.3 Experiments

We have carried out the experiments on the Large Biomedical Ontologies (*LargeBio*) track provided by the Ontology Alignment Evaluation Initiative (OAEI) campaign for the year 2020. *LargeBio* is composed of three independently developed large and semantically rich ontologies (*See* Table 2), namely *FMA* (Foundational Model of Anatomy), *NCI* (National Cancer Institute Thesaurus), and *SNOMED-CT* (Clinical Terms). OAEI provides reference alignments between each pair of the *LargeBio* ontologies based on the UMLS metathesaurus [62], namely *FMA-NCI, FMA-SNOMED* and *SNOMED-NCI* (*See* Table 3). *LargeBio* ontologies and reference alignments are downloadable from the OAEI^16^ website.

**Table 2:** Number of Entities in the *LargeBio* Ontologies.

LargeBio	Classes	Object Prop.	Data Prop.	Instances	Logical Axioms
FMA	78,988	0	54	0	79,218
NCI	66,724	123	67	0	96,046
SNOMED-CT	122,464	55	0	0	191,203
**Total**	268,176	178	121	0	366,467

**Table 3:** Number of correspondences in the *LargeBio* reference alignments.

Alignment	Original			Disambiguated		
	≡	?${}^{★}$	Total	≡	?${}^{★}$	Total
FMA-NCI	2,686	338	3,024	2,369	190	2,559
FMA-SNOMED	6,026	2,982	9,008	5,209	2,579	7,788
SNOMED-NCI	17,210	1,634	18,844	13,606	790	14,396
**Total Correspondences**	25,922${}^{††}$	4,954	30,876${}^{†}$	21,184${}^{‡‡}$	3,559	24,743${}^{‡}$

★ When these incoherence-causing correspondences are deleted, the alignment becomes repaired. † The *original* reference alignments contain 30,876 correspondences. †† The *repaired* reference alignments contain 25,922 correspondences. ‡ The *disambiguated* reference alignments contain 24,743 correspondences. ‡‡ The *disambiguated & repaired* reference alignments contain 21,184 correspondences.

In the OAEI reference alignments (*See* Table 3), correspondences having a relation flagged by the symbol ”≡” are correct *equivalence* correspondences; However, correspondences having a relation flagged by the symbol ”?” are correct *equivalence* correspondences involved in the introduction of unsatisfiable classes in the future integrated ontology.

We have integrated the three *LargeBio* ontologies FMA ($O_{1}$), NCI ($O_{2}$) and SNOMED-CT ($O_{3}$) using their three pairwise reference alignments *FMA-NCI, FMA-SNOMED* and *SNOMED-NCI*. The IRI of our output ontology is ”*http://integration*” for OIAR and ”*http://merging*” for AROM. All tests have been performed with a confidence threshold equal to 0.0, which means that we have not trimmed the input alignments; so, the input alignments still contain high and low confidence correspondences.

### 5.4 Examples of ontology integration cases using OIAR

#### 5.4.1 General case example

In this example, there are three *equivalence* correspondences extracted from *FMA-NCI, SNOMED-NCI* and *FMA-SNOMED* reference alignments, respectively, as follows (*See* Figure A.1):

$O_{1}$ #Skin_of_head ≡ $O_{2}$ #Head_Skin

$O_{3}$ #Skin_structure_of_head ≡ $O_{2}$ #Head_Skin

$O_{1}$ #Skin_of_head ≡ $O_{3}$ #Skin_structure_of_head

The first correspondence (shown in Figure A.1a) matches the class ”*Skin_of_head*” from *FMA* to the class ”*Head_Skin*” from *NCI*. The second correspondence (shown in Figure A.1b) matches the class ”*Skin_structure_of_head*” from *SNOMED* to the class ”*Head_Skin*” from *NCI*. And the third correspondence (shown in Figure A.1c) matches the class ”*Skin_of_head*” from *FMA* to the class ”*Skin_structure_of_head*” from *SNOMED*.

The definition/description of the class ”*Skin_of_head*” in its original ontology and in the output integrated ontology can be expressed in DL as follows:



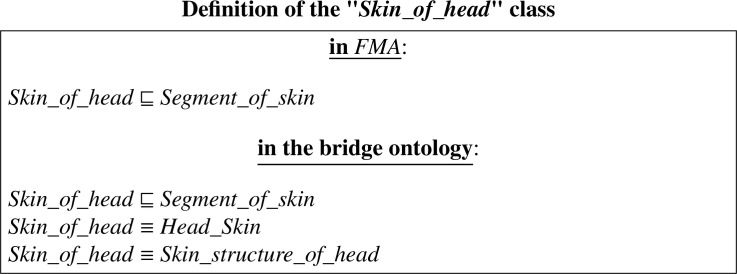



In Appendix A, we provide definitions written in RDF/XML^17^ which is the standard format/syntax for expressing ontologies. The definition of the class ”*Skin_of_head*” in its original ontology *FMA* is shown in Figure A.2. Figure A.3 shows an excerpt from the ontology that resulted from the integration of the *LargeBio* ontologies using OIAR. The framed axioms are the added *bridging* axioms translating the *equivalence* correspondences of the input alignments. In the non-refactored version of OIAR (*See* Figure A.3a), axioms of the integrated ontology are exactly like the original ones. However, in the refactored version of OIAR (*See* Figure A.3b), axioms of the integrated ontology are exactly like the original ones, except that the IRIs of all the mentioned entities are customized.

#### 5.4.2 Alignment disambiguation example

In this example, we focus on the case where there are ambiguous correspondences in the input alignments. There are three *equivalence* correspondences extracted from *FMA-NCI* and *FMA-SNOMED* reference alignments (*See* Figure A.4). They match the class ”*Abdominal_lymph_node*” (from *FMA*) with three other classes (from *NCI* and *SNOMED*) as follows:

*Abdominal_lymph_node* (from *FMA*) is equivalent to:

≡ *Intra-abdominal_Lymph_Node* (*NCI*) [0.50]
≡ *Abdominal_lymph_node_structure* (*SNOMED*) [0.61]
≡ *Abdominal_lymph_node_group* (*SNOMED*) [0.55]

where ”*Abdominal_lymph_node_group*” is a subclass of ”*Abdominal_lymph_node_structure*”. Values into brackets are the confidence/similarity values of these correspondences.

The first correspondence that matches ”*Abdominal_lymph_node*” with ”*Intra-abdominal_Lymph_Node*” has a similarity measure of 0.5 (*See* Figure A.4a). The second correspondence that matches ”*Abdominal_lymph_node*” with ”*Abdominal_lymph_node_structure*” has a similarity measure of 0.61 (*See* Figure A.4b). And the third correspondence that matches ”*Abdominal_lymph_node*” to ”*Abdominal_lymph_node_group*” has a similarity measure equal to 0.55 (*See* Figure A.4c). Notice that the second and third correspondences are ambiguous correspondences because the same *source* class ”*Abdominal_lymph_node*” coming from *FMA* is matched to two *target* classes coming from *SNOMED-CT*. The second correspondence is more reliable than the third one because it has a higher similarity value. Notice that if the alignment disambiguation step is executed, then the third correspondence will be removed. The disambiguation algorithm only keeps the highest ambiguous correspondence, which is in our case the second correspondence, in order to obtain a 1-to-1 input alignment.

The definition of the class ”*Abdominal_lymph_node*” in its original ontology and in the output integrated ontology can be expressed in DL as follows:



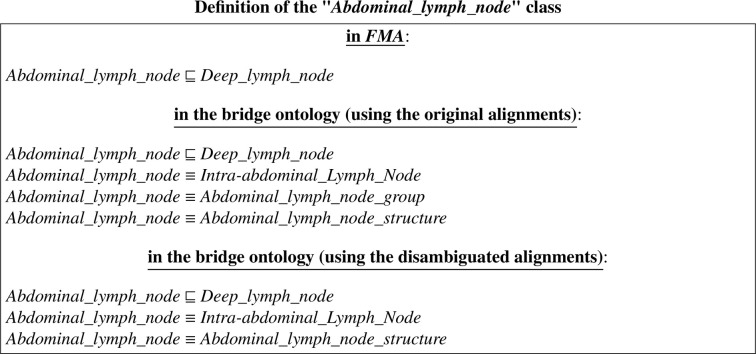



In Appendix A, we provide definitions written in the RDF/XML syntax. The definition of the class ”*Abdominal_lymph_node*” in its original ontology *FMA* is shown in Figure A.5. Figure A.6 shows the definition of the class ”*Abdominal_lymph_node*” in the bridge ontology that resulted from the integration of the *LargeBio* ontologies using the original reference alignments. Whereas, Figure A.7 shows the definition of the class ”*Abdominal_lymph_node*” in the resulting bridge ontology using the disambiguated reference alignments (*i.e.*, after disambiguating the input alignments). Therefore, Figure A.6 contains three equivalence *bridging* axioms, while Figure A.7 only contains two equivalence *bridging* axioms, because the third correspondence was removed during the disambiguation step.

#### 5.4.3 Incoherence example

Integrating the *LargeBio* ontologies introduces many unsatisfiable classes in the resulting integrated ontology. In OWL, an *equivalence* axiom linking two classes is formally and implicitly equal to two *subsumption* axioms in both directions, as stated in Eq. 1 where $C_{1}$ and $C_{2}$ are two classes.

(1) $$\langle C_{1},C_{2},\equiv \rangle =\langle C_{1},C_{2},⊑\rangle +\langle C_{2},C_{1},⊑\rangle$$

The addition of these implicit subsumption (<) relations will alter the structure of the input ontologies and will infer new knowledge that may be contradictory, mainly because of the existence of disjoint axioms coming from the input ontologies.

Example 1 **Coherence Violation**

**Fig. 7:**
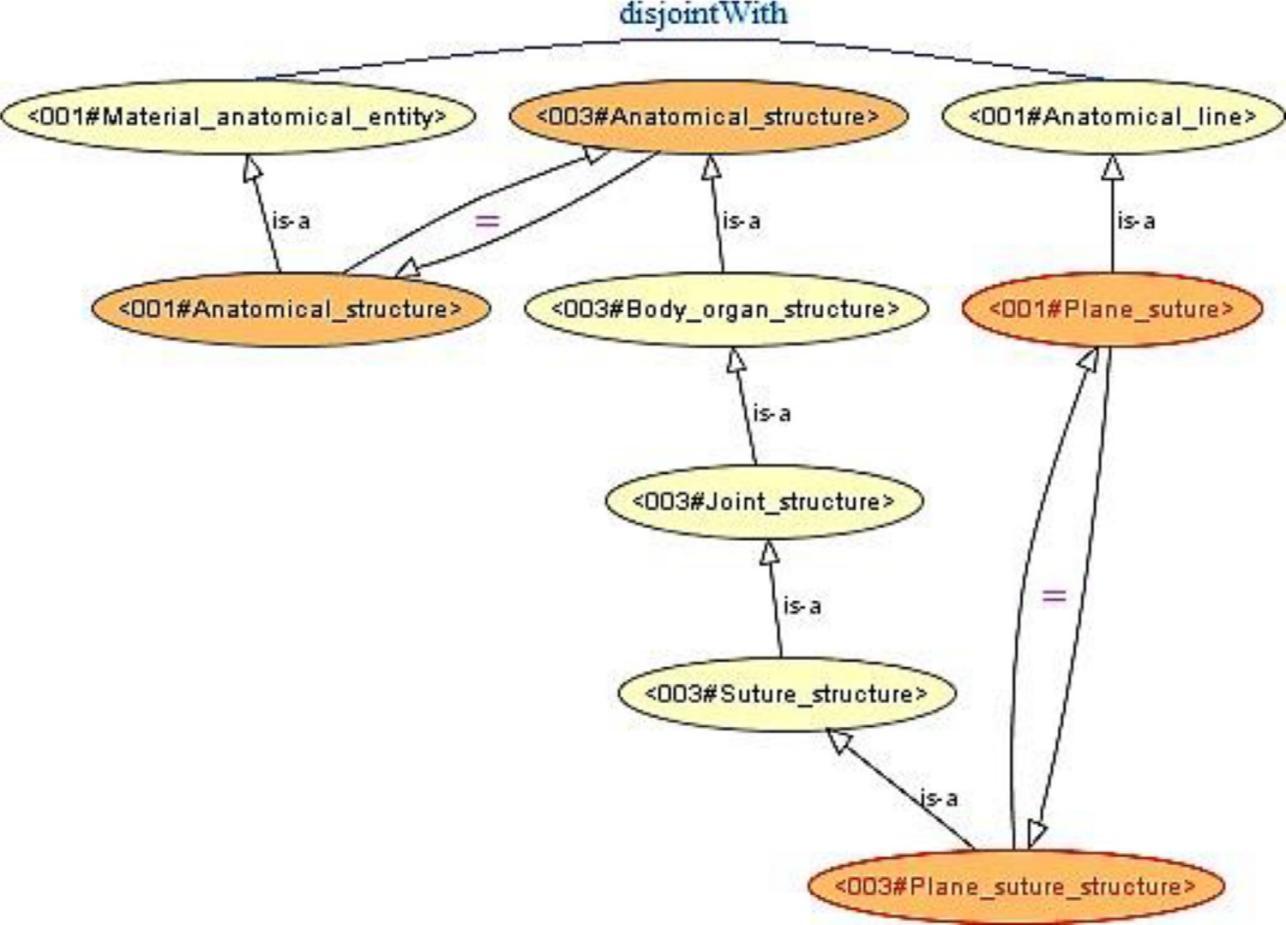
Unsatisfiable class formation in a bridge ontology.

Fig. 7 shows an example of two unsatisfiable classes in the integrated ontology, which are ”*001#Plane_suture*” and ”*003#Plane_suture_structure*” originating from FMA ($O_{1}$) and SNOMED ($O_{3}$), respectively. In Fig. 7, we omit IRI prefixes of the entities for readability reasons. When adding the two *equivalence* axioms linking ”*001#Plane_suture*” and ”*003#Plane_suture_structure*” and linking ”*001#Anatomical_structure*” and ”*003#Anatomical_structure*”, each of these *equivalence* axioms becomes a set of two reciprocal *subsumption* axioms. By inference, the two classes ”*001#Plane_suture*” and ”*003#Plane_suture_structure*” (circled in red) become sub-classes of ”*001#Material_anatomical_entity*” and ”*001#Anatomical_line*” which are two disjoint classes from *FMA*. These two classes are unsatisfiable because a class can never be a subclass of two disjoint classes. To ensure the coherence of the integrated ontology, we will face a dilemma between sacrificing an *equivalence* correspondence from the alignment (by removing the correspondence linking ”*001#Plane_suture*” and ”*003#Plane_suture_structure*” in our case), which will introduce redundant entities and reduce interoperability between input ontologies, or sacrificing the *disjointness* axiom from the input ontology *FMA*, which will be a knowledge loss. Ontology matching and alignment repair are beyond the scope of this paper. An alignment repair process would remove all the correspondences that cause unsatisfiable entities in the integrated ontology.

### 5.5 Examples of ontology integration cases using AROM

#### 5.5.1 General case example

We take the same example that we have done for OIAR (*See* Example 5.4.1), but we perform a full merge instead of a simple merge. The correspondences (of Figure A.1) will lead to the merging of the three matched classes: ”*Skin_of_head*”, ”*Head_Skin*” and ”*Skin_structure _of_head*” because they are mentioned as equivalent classes in the input alignments. The definition/description of these three matched classes (in their original ontologies *FMA, NCI* and *SNOMED*) can be expressed in DL as follows (*See* Figure B.1):



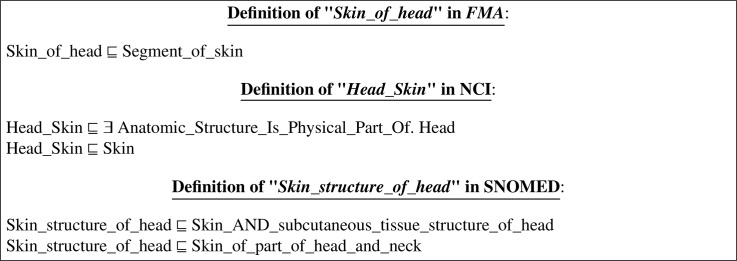



The three equivalent classes have been fully merged into a single class in our output merged ontology. The merged class:

• is identified by the short name ”*Code_19351*”;
• has three added labels (framed in red), which are actually the short names of the classes that have been merged into it (We attach each short name to its ontology number (ID) to directly see from which ontology it originates);
• and captures all the knowledge of the three equivalent classes that have been merged.

The definition of the merged class ”*Code_19351*” in the output merged ontology can be expressed in DL as follows:



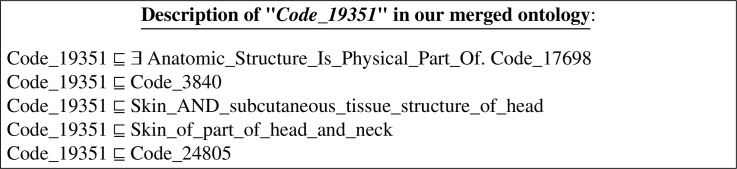



Notice that all the axioms describing the equivalent entities were preserved in the merged entity ”*Code_19351*”; and all the merged entities, mentioned in the description of ”*Code_19351*”, are also identified by their corresponding unique codes (as short names). That is, the class ”*Segment_of_skin*” (from *FMA*) was merged with its equivalent classes (from *NCI* and *SNOMED*) to form the class ”*000#Code_3840*”; The class ”*Skin*” (from *NCI*) was merged with its equivalent classes to form the class ”*000#Code_24805*”; And the class ”*Head*” (from *NCI*) was merged with its equivalent classes to form the class ”*000#Code_17698*”.

In Appendix B, we provide definitions written in RDF/XML. Figure B.2 shows an excerpt from the ontology that results from merging the three *LargeBio* ontologies using AROM. In the non-refactored version of AROM (*See* Figure B.2a), axioms of the merged ontology are exactly like the original ones. However, in the refactored version of AROM (*See* Figure B.2b), axioms of the merged ontology are exactly like the original ones, except that the IRIs of all the mentioned entities are customized.

#### 5.5.2 Incoherence examples

Example 2 **Coherence Violation**

**Fig. 8:**
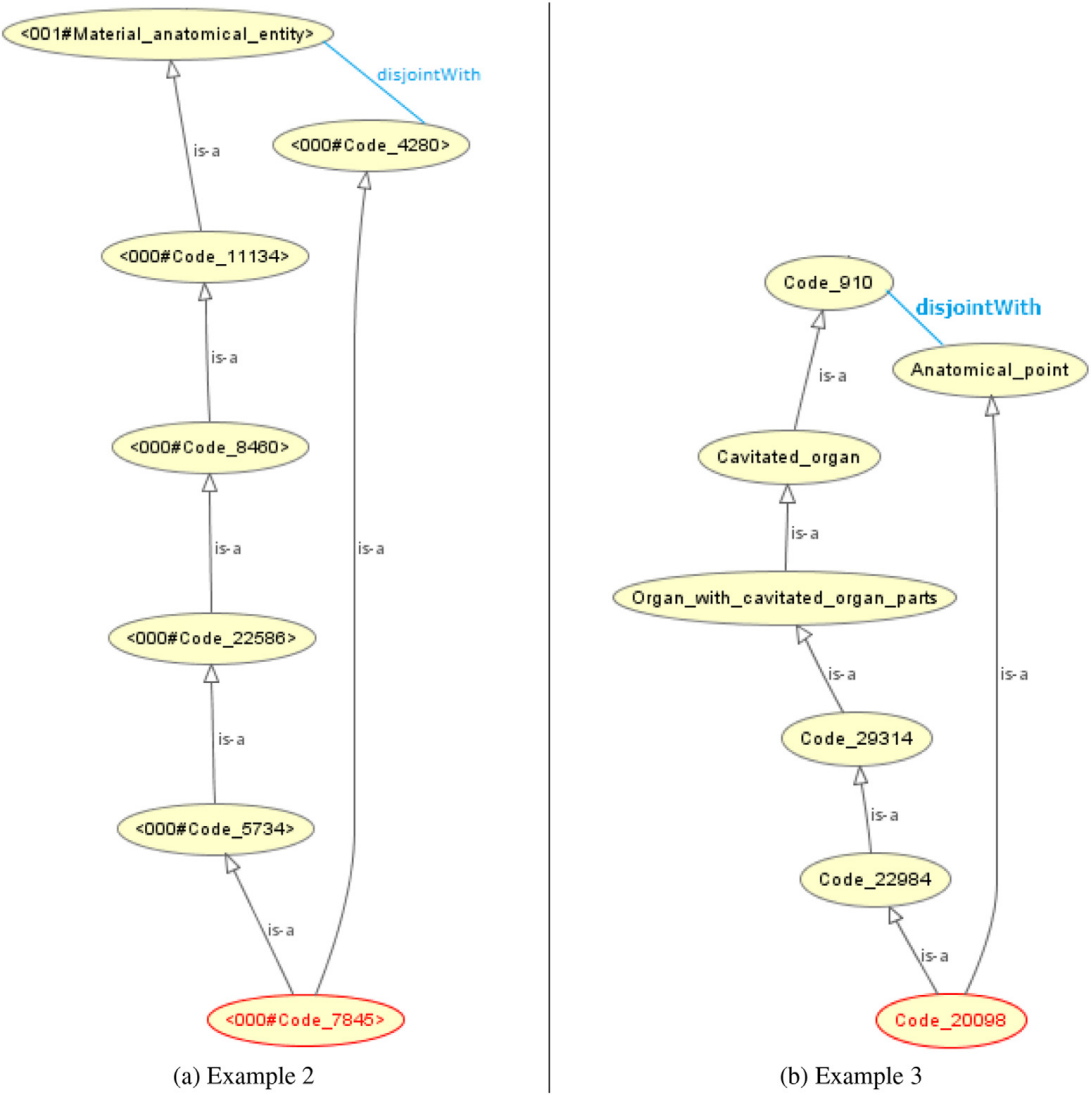
Examples of an Unsatisfiable Class Formation in a Full-Merge Ontology.

In Fig. 8a, we take the same incoherence example that we have done for OIAR (*See* Example 1), but we perform a full merge instead of a simple merge. Figs. 8a and B.3 show an unsatisfiable class in the merged ontology, which is the class ”*000#Code_7845*” circled in red. In Fig. 8a, we omit IRI prefixes of the entities for readability reasons. After merging the equivalent classes ”*Plane_suture*” belonging to *FMA* ($O_{1}$) and ”*Plane_suture_structure*” belonging to *SNOMED* ($O_{3}$), we get the merged class ”*000#Code_7845*”. After merging the equivalent classes ”*Anatomical_structure*” belonging to *FMA* ($O_{1}$) and ”*Anatomical_structure*” belonging to *SNOMED* ($O_{3}$), we get the merged class ”*000#Code_11134*”. By inference, the merged class ”*000#Code_7845*” becomes a subclass of ”*001#Material_anatomical_entity*” and ”*001#Anatomical_line*”, which are two disjoint classes coming from *FMA* ($O_{1}$). After the merge, ”*001#Anatomical_line*” is merged with its equivalent entities to form the class ”*000#Code_4280*”. Similarly, ”*003#Suture_structure*”, ”*003#Joint_structure*” and ”*003#Body_organ_structure*” were merged with their corresponding equivalent classes to form the classes ”*000#Code_5734*”, ”*000#Code_22586*” and ”*000#Code_8460*”, respectively.

**Algorithm 1:**
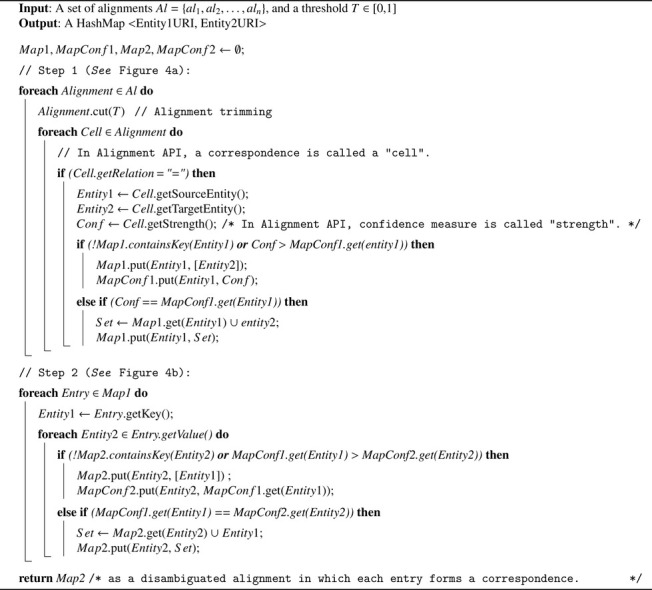
Alignment Disambiguation.

Example 3 **Coherence Violation**

Figs. 8b and B.4 show another example of an unsatisfiable class in the merged ontology, which is the class ”*000#Code_20098*” circled in red. In Fig. 8b, we omit IRI prefixes of the entities for readability reasons. After merging the equivalent classes ”*Apex_of_heart*” belonging to *FMA* ($O_{1}$), ”*Apex_of_the_Heart*” belonging to *NCI* ($O_{2}$), and ”*Structure_of_apex_of_heart*” belonging to *SNOMED* ($O_{3}$), the merged class ”*000#Code_20098*” becomes, by inference, a subclass of ”*001#Organ*” and ”*001#Anatomical_point*” which are two disjoint classes coming from *FMA* ($O_{1}$). After the merge, ”*001#Organ*” was merged with its equivalent class ”*002#Organ*” (from *NCI*) to form the class ”*000#Code_910*”.

### 5.6 Results

Tables 4 and 5 sketch the characteristics of ontologies resulting from the integration of *LargeBio* ontologies using OIAR and AROM, respectively. We have performed four runs using OIAR, and four runs using AROM. In each run, we change the type of the input alignments; Alignments can be original (unaltered), disambiguated, repaired, or disambiguated and repaired. In Tables 4 and 5:

1. The *Original* column means that we have used all correspondences from the input alignments (*i.e.*, we have kept the input alignments ambiguous and unrepaired);
2. The *Disambiguated* column means that we have used all correspondences from the disambiguated input alignments (*i.e.*, we have performed the alignments’ disambiguation step that only keeps one correspondence from each set of ambiguous correspondences);
3. The *Repaired* column means that we have only used correspondences having relations ”≡” from the input alignments (*i.e.*, we have repaired the input alignments by removing their correspondences that have a relation ”?”), and;
4. The *Disambiguated & Repaired* column means that we have only used the correspondences having relations ”≡” from the disambiguated input alignments (*i.e.*, we have disambiguated and repaired the input alignments).

**Table 4:** Characteristics of the ontology resulting from the integration of *LargeBio* ontologies using OIAR.

**Simple-Merge Integrated Ontology Features**	Input Alignments			
	Original	Repaired	Disambiguated	Disambiguated & Repaired
Classes	268,176			
Object Properties	178			
Datatype Properties	121			
Instances	0			
Logical Axioms^a^	397,343 (366,467 +30,876)	392,389 (366,467 +25,922)	391,210 (366,467 +24,743)	387,651 (366,467 +21,184)
Consistency^b^	✓	✓	✓	✓
Unsatisfiable Classes^b^	203,675	49,046	155,775	43,078
Redundant Classes	0	8,498	4,501	10,540
Runtime^c^ (min)	0.70	0.68	0.71	0.70

a In parentheses, 366,467 is the total number of axioms of all input ontologies, and the value after the sum operator is the total number of correspondences of all input alignments.

b Computed using the ELK ontology reasoner [59].

c Runtimes do not include matching times, since we take pre-established alignments as input.

**Table 5:** Characteristics of the ontology resulting from the integration of *LargeBio* ontologies using AROM.

**Full-Merge Integrated Ontology Features**	Input Alignments			
	Original	Repaired	Disambiguated	Disambiguated & Repaired
Classes	240,634	244,173	245,334	248,097
Object Properties	178			
Datatype Properties	121			
Instances	0			
Logical Axioms	359,600	360,577	362,404	363,135
Consistency^a^	✓	✓	✓	✓
Unsatisfiable Classes^a^	177,975	42,450	138,523	38,067
Redundant Classes	0	8,498	4,501	10,540
Runtime^b^ (min)	0.76	0.75	0.72	0.77

a Computed using the ELK ontology reasoner [59].

b Runtimes do not include matching times nor alignment repair times.

### 5.7 Observations and discussion

OIAR and AROM results are complete in the sense that they conserve all entities and axioms from the input ontologies. By observing the four columns of Tables 4 and 5, we notice that the quality of the integrated ontology depends on the quality of the input alignments (*i.e.*, it depends on the performance of the ontology matching module and whether or not an alignment repair step is included in the matching process).

Unsatisfiable entities, identified by the ELK reasoner, are formed because of the heterogeneous conceptualizations of the input ontologies. Indeed, the input ontologies may be in disagreement with each other because they have incompatible organizations/structuring or contradictory descriptions of the same entities. This leads to the incoherence of the integrated ontology. Unsatisfiable classes are often repaired by removing the incoherence-causing correspondences. However, removing correct correspondences generates redundant (or duplicated) classes. The latter are distinct but actually equivalent classes coming from different input ontologies. We conclude that the requirements of *ontology coherence* and *minimality* (*i.e., entity non-redundancy*) can never be both fulfilled at the same time.

Although the alignment disambiguation removes more correspondences than does the alignment repair (as shown in Table 3), the use of the disambiguated alignments generates much less redundant classes in the integrated ontology than the use of the repaired alignments. This is because the ambiguous correspondences have so many entities in common (*i.e.*, where the same entities appear in many correspondences). Thus, after removing these ambiguous correspondences by the disambiguation process, the number of redundant classes will be much less than expected, because classes composing the removed correspondences have a high overlap.

It is important to mention that if we integrate the three *LargeBio* ontologies using all correspondences from the original (unrepaired and ambiguous) alignments and without conserving any *DisjointWith* axiom from the input ontologies, we do not get any unsatisfiable class in our integrated ontology. In this case, our integrated ontology is coherent but incomplete, *i.e.*, lacking valuable disjoint knowledge. This proves that *disjointness* axioms are the major cause of semantic conflicts in the integration of *LargeBio* ontologies. Therefore, in case we wish to conserve the *disjointness* axioms of the input ontologies (like in our case here), our integrated ontology needs to be repaired. We conclude that the requirements of *ontology coherence* and *ontology knowledge preservation* can never be both fulfilled at the same time.

It should be noted that the *full merge* ontology always generates fewer unsatisfiable entities than does the *simple merge* ontology because it naturally contains fewer entities after they have been fully merged. Nevertheless, performing a *full merge* or a *simple merge* is exactly the same from a semantic/logical point of view. In fact, if one leads to unsatisfiable entities, then the other will do so; and if one does not lead to unsatisfiable entities, the other will do so.

We also notice that if we integrate two *LargeBio* ontologies using the repaired reference alignment between them, then we get a consistent and coherent ontology (*i.e.*, that has no unsatisfiable classes). However, if we integrate the three *LargeBio* ontologies using the three repaired pairwise alignments (between ontology pairs), then we get an incoherent ontology that has considerable unsatisfiable classes. We conclude that in an integration of multiple ontologies using pairwise alignments, we cannot escape unsatisfiability even though we use repaired alignments. These unsatisfiable classes are beyond the abilities of the current alignment repair systems. Actually, alignment repair systems are dedicated to only integrating two ontologies using a pairwise alignment between them; they do not deal with the simultaneous integration of multiple ontologies. This underscores the compelling need for alignment repair systems that could deal with the holistic ontology integration using pairwise alignments.

Still, OIAR and AROM can considerably help ontology developers in making initial ontology integration steps, since they reduce the time and cost required for conceptualizing the ontology domain from scratch. Runtimes of the complete process of both algorithms do not exceed one minute for integrating the *LargeBio* ontologies. Remember that OIAR and AROM take as input external alignments—that can be repaired or not; Thus, runtimes do not include neither *ontology matching* times nor *alignment repairing* times.

Finally, as shown in Table 3, the *disambiguation* step removed more correspondences from the original alignments than did the *repair* step. Therefore, we expect that the integration of the *LargeBio* ontologies using the disambiguated alignments will generate less unsatisfiable classes than the one using the repaired alignments. However, contrary to our expectations, when comparing the two columns ”*Disambiguated* alignments” and ”*Repaired* alignments” from Tables 4 and 5, we observe that the use of the disambiguated alignments generates much more unsatisfiable classes than the use of the repaired alignments. We deduce that the alignment disambiguation is an ”aggressive” approach that removes unnecessary correspondences without being able to guarantee coherence in the integrated ontology. We should note that the repaired alignments do contain ambiguous correspondences (as shown in Table 3). Despite being ambiguous, when we use the *LargeBio* repaired reference alignments to integrate each ontology pair separately, we do not get any unsatisfiable classes in the integrated ontology. In other terms, when we integrate two *LargeBio* ontologies using the repaired reference alignment between them, the integrated ontology is always consistent and coherent. Here, disambiguating the repaired alignment is useless and will indeed generate many redundant classes (that should have been merged or linked by an *equivalence* axiom) in the integrated ontology. To sum up, bear in mind that not all ambiguous *equivalence* correspondences generate unsatisfiable entities in the integrated ontology. However, the alignment repair approaches may include an alignment disambiguation step in some cases whenever needed.

## 6 Conclusion

We have proposed a holistic method to support the simultaneous and customized integration of multiple ontologies by relying on external alignments. As a result, we have generated a comprehensive freely-available software framework that includes the different implementations. As extensively discussed in the paper, such implementations reflect different integration strategies (*i.e.*, simple/full merge) to provide different kinds of outcome (*e.g.*, refactored/non-refactored integrated ontology). Additionally, we have validated and tested the proposed framework by running several experiments based on well-known ontologies, and we have discussed the results in context and against our initial assumptions. At a theoretical level, results are in line with expectations, as major limitations—*i.e.*, ontology inconsistency or unsatisfiabilities generated by contradictory specifications—are commonly considered as open research issues within the community [63].

Overall, we believe that the holistic approach does not introduce specific inconveniences along the integration process, while it may significantly contribute to create a more usable and practical integration environment.

## Declaration of Competing Interest

The authors declare that they have no known competing financial interests or personal relationships that could have appeared to influence the work reported in this paper.

## Appendix A Supplementary materials

Supplementary Data S1 Supplementary Raw Research Data. This is open data under the CC BY license http://creativecommons.org/licenses/by/4.0/ Supplementary Data S1

## Footnotes

1 https://dbs.uni-leipzig.de/Research/coma.html

2 http://ws.nju.edu.cn/falcon-ao/

3 https://github.com/ernestojimenezruiz/logmap-matcher

4 http://yamplusplus.lirmm.fr/matcher

5 https://github.com/AgreementMakerLight/AML-Project

6 https://www.w3.org

7 http://oaei.ontologymatching.org/2017/conference/index.html

8 The Alignment API format is the most consensual ontology alignment format. It represents a set of simple binary semantic correspondences between entities coming from two ontologies.

9 https://github.com/inesosman/OIAR

10 https://github.com/owlcs/owlapi/wiki/Documentation

11 https://github.com/inesosman/AROM

16 http://oaei.ontologymatching.org/

17 https://www.w3.org/TR/rdf-syntax-grammar/

## Footnotes

12 https://github.com/owlcs/owlapi/wiki

13 http://alignapi.gforge.inria.fr/

14 http://www.hermit-reasoner.com/

15 https://www.cs.ox.ac.uk/isg/tools/ELK/
