# Supplementary material for: An Alignment-Based Implementation of a Holistic Ontology Integration Method
Source: MethodsX. 2021 Jul 23;8:101460. doi: 10.1016/j.mex.2021.101460 (PMC8374672; doi:10.1016/j.mex.2021.101460)
Supplement: Supplementary Data S1 — Supplementary Raw Research Data. This is open data under the CC BY license http://creativecommons.org/licenses/by/4.0/ [file mmc1.pdf]

## Supplementary Material

*Appendix A. Figures of the OIAR Experimentation*

```
<Cell>
    <entity1 rdf:resource="http://bioontology.org/projects/ontologies/fma/fmaOwlDlComponent_2_0#Skin_of_head"/>
    <entity2 rdf:resource="http://ncicb.nci.nih.gov/xml/owl/EVS/Thesaurus.owl#Head_Skin"/>
    <measure rdf:datatype="http://www.w3.org/2001/XMLSchema#float">0.67</measure>
    <relation>=</relation>
</Cell>
```

(a) from FMA-NCI

```
<Cell>
    <entity1 rdf:resource="http://www.ihtsdo.org/snomed#Skin_structure_of_head"/>
    <entity2 rdf:resource="http://ncicb.nci.nih.gov/xml/owl/EVS/Thesaurus.owl#Head_Skin"/>
    <measure rdf:datatype="http://www.w3.org/2001/XMLSchema#float">0.35</measure>
    <relation>=</relation>
</Cell>
```

(b) from SNOMED-NCI

```
<Cell>
    <entity1 rdf:resource="http://bioontology.org/projects/ontologies/fma/fmaOwlDlComponent_2_0#Skin_of_head"/>
    <entity2 rdf:resource="http://www.ihtsdo.org/snomed#Skin_structure_of_head"/>
    <measure rdf:datatype="http://www.w3.org/2001/XMLSchema#float">0.47</measure>
    <relation>?</relation>
</Cell>
```

(c) from FMA-SNOMED

Figure A.1: Equivalence Correspondences from the Reference Alignments

```
<!-- http://bioontology.org/projects/ontologies/fma/fmaOwlDlComponent_2_0#Skin_of_head -->

<owl:Class rdf:about="http://bioontology.org/projects/ontologies/fma/fmaOwlDlComponent_2_0#Skin_of_head">
    <rdfs:label xml:lang="en">Head skin</rdfs:label>
    <rdfs:label xml:lang="en">Skin of head</rdfs:label>
    <rdfs:subClassOf rdf:resource="http://bioontology.org/projects/ontologies/fma/fmaOwlDlComponent_2_0#Segment_of_skin"/>
</owl:Class>
```

Figure A.2: Description of the Class "*Skin_of_head*" in FMA

```xml
<!-- http://bioontology.org/projects/ontologies/fma/fmaOwlDlComponent_2_0#Skin_of_head -->

<owl:Class rdf:about="http://bioontology.org/projects/ontologies/fma/fmaOwlDlComponent_2_0#Skin_of_head">
    <rdfs:label xml:lang="en">Head skin</rdfs:label>
    <rdfs:label xml:lang="en">Skin of head</rdfs:label>
    <owl:equivalentClass rdf:resource="http://ncicb.nci.nih.gov/xml/owl/EVS/Thesaurus.owl#Head_Skin"/>
    <owl:equivalentClass rdf:resource="http://www.ihtsdo.org/snomed#Skin_structure_of_head"/>
    <rdfs:subClassOf rdf:resource="http://bioontology.org/projects/ontologies/fma/fmaOwlDlComponent_2_0#Segment_of_skin"/>
</owl:Class>
```

(a) Non-Refactored Version

```xml
<!-- http://integration/001#Skin_of_head -->

<owl:Class rdf:about="http://integration/001#Skin_of_head">
    <rdfs:label xml:lang="en">Head skin</rdfs:label>
    <rdfs:label xml:lang="en">Skin of head</rdfs:label>
    <owl:equivalentClass rdf:resource="http://integration/002#Head_Skin"/>
    <owl:equivalentClass rdf:resource="http://integration/003#Skin_structure_of_head"/>
    <rdfs:subClassOf rdf:resource="http://integration/001#Segment_of_skin"/>
</owl:Class>
```

(b) Refactored Version

Figure A.3: Description of the Class "*Skin_of_head*" in the Bridge Ontology Resulting from the *LargeBio* Integration using OIAR

```xml
<Cell>
    <entity1 rdf:resource="http://bioontology.org/projects/ontologies/fma/fmaOwlDlComponent_2_0#Abdominal_lymph_node"/>
    <entity2 rdf:resource="http://ncicb.nci.nih.gov/xml/owl/EVS/Thesaurus.owl#Intra-abdominal_Lymph_Node"/>
    <measure rdf:datatype="http://www.w3.org/2001/XMLSchema#float">0.5</measure>
    <relation>=</relation>
</Cell>
```

(a) from FMA-NCI

```xml
<Cell>
    <entity1 rdf:resource="http://bioontology.org/projects/ontologies/fma/fmaOwlDlComponent_2_0#Abdominal_lymph_node"/>
    <entity2 rdf:resource="http://www.ihtsdo.org/snomed#Abdominal_lymph_node_structure"/>
    <measure rdf:datatype="http://www.w3.org/2001/XMLSchema#float">0.61</measure>
    <relation>=</relation>
</Cell>
```

(b) from FMA-SNOMED

```xml
<Cell>
    <entity1 rdf:resource="http://bioontology.org/projects/ontologies/fma/fmaOwlDlComponent_2_0#Abdominal_lymph_node"/>
    <entity2 rdf:resource="http://www.ihtsdo.org/snomed#Abdominal_lymph_node_group"/>
    <measure rdf:datatype="http://www.w3.org/2001/XMLSchema#float">0.55</measure>
    <relation>=</relation>
</Cell>
```

(c) from FMA-SNOMED

Figure A.4: Equivalence Correspondences from the Reference Alignments

```xml
<!-- http://bioontology.org/projects/ontologies/fma/fmaOwlDlComponent_2_0#Abdominal_lymph_node -->

<owl:Class rdf:about="http://bioontology.org/projects/ontologies/fma/fmaOwlDlComponent_2_0#Abdominal_lymph_node">
    <rdfs:label xml:lang="en">Abdominal lymph node</rdfs:label>
    <rdfs:label xml:lang="en">Lymph node of abdomen</rdfs:label>
    <rdfs:subClassOf rdf:resource="http://bioontology.org/projects/ontologies/fma/fmaOwlDlComponent_2_0#Deep_lymph_node"/>
</owl:Class>
```

Figure A.5: Description of the Class "*Abdominal_lymph_node*" in FMA

```xml
<!-- http://integration/001#Abdominal_lymph_node -->

<owl:Class rdf:about="http://integration/001#Abdominal_lymph_node">
    <rdfs:label xml:lang="en">Abdominal lymph node</rdfs:label>
    <rdfs:label xml:lang="en">Lymph node of abdomen</rdfs:label>
    <owl:equivalentClass rdf:resource="http://integration/002#Intra-abdominal_Lymph_Node"/>
    <owl:equivalentClass rdf:resource="http://integration/003#Abdominal_lymph_node_group"/>
    <owl:equivalentClass rdf:resource="http://integration/003#Abdominal_lymph_node_structure"/>
    <rdfs:subClassOf rdf:resource="http://integration/001#Deep_lymph_node"/>
</owl:Class>
```

(a) Non-Refactored Version

```xml
<!-- http://bioontology.org/projects/ontologies/fma/fmaOwlDlComponent_2_0#Abdominal_lymph_node -->

<owl:Class rdf:about="http://bioontology.org/projects/ontologies/fma/fmaOwlDlComponent_2_0#Abdominal_lymph_node">
    <rdfs:label xml:lang="en">Abdominal lymph node</rdfs:label>
    <rdfs:label xml:lang="en">Lymph node of abdomen</rdfs:label>
    <owl:equivalentClass rdf:resource="http://ncicb.nci.nih.gov/xml/owl/EVS/Thesaurus.owl#Intra-abdominal_Lymph_Node"/>
    <owl:equivalentClass rdf:resource="http://www.ihtsdo.org/snomed#Abdominal_lymph_node_group"/>
    <owl:equivalentClass rdf:resource="http://www.ihtsdo.org/snomed#Abdominal_lymph_node_structure"/>
    <rdfs:subClassOf rdf:resource="http://bioontology.org/projects/ontologies/fma/fmaOwlDlComponent_2_0#Deep_lymph_node"/>
</owl:Class>
```

(b) Refactored Version

Figure A.6: Description of the Class "*Abdominal_lymph_node*" in the Bridge Ontology Generated by OIAR using the Original Input Alignments

```xml
<!-- http://bioontology.org/projects/ontologies/fma/fmaOwlDlComponent_2_0#Abdominal_lymph_node -->

<owl:Class rdf:about="http://bioontology.org/projects/ontologies/fma/fmaOwlDlComponent_2_0#Abdominal_lymph_node">
    <rdfs:label xml:lang="en">Abdominal lymph node</rdfs:label>
    <rdfs:label xml:lang="en">Lymph node of abdomen</rdfs:label>
    <owl:equivalentClass rdf:resource="http://ncicb.nci.nih.gov/xml/owl/EVS/Thesaurus.owl#Intra-abdominal_Lymph_Node"/>
    <owl:equivalentClass rdf:resource="http://www.ihtsdo.org/snomed#Abdominal_lymph_node_structure"/>
    <rdfs:subClassOf rdf:resource="http://bioontology.org/projects/ontologies/fma/fmaOwlDlComponent_2_0#Deep_lymph_node"/>
</owl:Class>
```

(a) Non-Refactored Version

```xml
<!-- http://integration/001#Abdominal_lymph_node -->

<owl:Class rdf:about="http://integration/001#Abdominal_lymph_node">
    <rdfs:label xml:lang="en">Abdominal lymph node</rdfs:label>
    <rdfs:label xml:lang="en">Lymph node of abdomen</rdfs:label>
    <owl:equivalentClass rdf:resource="http://integration/002#Intra-abdominal_Lymph_Node"/>
    <owl:equivalentClass rdf:resource="http://integration/003#Abdominal_lymph_node_structure"/>
    <rdfs:subClassOf rdf:resource="http://integration/001#Deep_lymph_node"/>
</owl:Class>
```

(b) Refactored Version

Figure A.7: Description of the Class "*Abdominal_lymph_node*" in the Bridge Ontology Generated by OIAR using the Disambiguated Input Alignments

```
<!-- http://bioontology.org/projects/ontologies/fma/fmaOwlDlComponent_2_0#Skin_of_head -->

<owl:Class rdf:about="http://bioontology.org/projects/ontologies/fma/fmaOwlDlComponent_2_0#Skin_of_head">
    <rdfs:label xml:lang="en">Head skin</rdfs:label>
    <rdfs:label xml:lang="en">Skin of head</rdfs:label>
    <rdfs:subClassOf rdf:resource="http://bioontology.org/projects/ontologies/fma/fmaOwlDlComponent_2_0#Segment_of_skin"/>
</owl:Class>
```

(a) from FMA

```
<!-- http://ncicb.nci.nih.gov/xml/owl/EVS/Thesaurus.owl#Head_Skin -->

<owl:Class rdf:about="&Thesaurus;Head_Skin">
    <rdfs:label xml:lang="en">Head Skin</rdfs:label>
    <rdfs:subClassOf rdf:resource="&Thesaurus;Skin"/>
    <rdfs:subClassOf>
        <owl:Restriction>
            <owl:onProperty rdf:resource="&Thesaurus;Anatomic_Structure_Is_Physical_Part_Of"/>
            <owl:someValuesFrom rdf:resource="&Thesaurus;Head"/>
        </owl:Restriction>
    </rdfs:subClassOf>
</owl:Class>
```

(b) from NCI

```
<!-- http://www.ihtsdo.org/snomed#Skin_structure_of_head -->

<owl:Class rdf:about="&snomed;Skin_structure_of_head">
    <rdfs:label xml:lang="en">Skin_structure_of_head</rdfs:label>
    <rdfs:subClassOf rdf:resource="&snomed;Skin_AND_subcutaneous_tissue_structure_of_head"/>
    <rdfs:subClassOf rdf:resource="&snomed;Skin_of_part_of_head_and_neck"/>
</owl:Class>
```

(c) from SNOMED

Figure B.1: Description of the Equivalent Classes to be Merged

```
<!-- http://merging/000#Code_19351 -->

<owl:Class rdf:about="http://merging/000#Code_19351">
    <rdfs:label rdf:datatype="http://www.w3.org/2001/XMLSchema#string">1. Skin_of_head</rdfs:label>
    <rdfs:label rdf:datatype="http://www.w3.org/2001/XMLSchema#string">2. Head_Skin</rdfs:label>
    <rdfs:label rdf:datatype="http://www.w3.org/2001/XMLSchema#string">3. Skin_structure_of_head</rdfs:label>
    <rdfs:label xml:lang="en">Head Skin</rdfs:label>
    <rdfs:label xml:lang="en">Head skin</rdfs:label>
    <rdfs:label xml:lang="en">Skin of head</rdfs:label>
    <rdfs:label xml:lang="en">Skin_structure_of_head</rdfs:label>
    <rdfs:subClassOf rdf:resource="http://merging/000#Code_24805"/>
    <rdfs:subClassOf rdf:resource="http://merging/000#Code_3840"/>
    <rdfs:subClassOf rdf:resource="http://merging/003#Skin_AND_subcutaneous_tissue_structure_of_head"/>
    <rdfs:subClassOf rdf:resource="http://merging/003#Skin_of_part_of_head_and_neck"/>
    <rdfs:subClassOf>
        <owl:Restriction>
            <owl:onProperty rdf:resource="http://merging/002#Anatomic_Structure_Is_Physical_Part_Of"/>
            <owl:someValuesFrom rdf:resource="http://merging/000#Code_17698"/>
        </owl:Restriction>
    </rdfs:subClassOf>
</owl:Class>
```

(a) Non-Refactored Version

```
<!-- http://merging#Code_19351 -->

<owl:Class rdf:about="http://merging#Code_19351">
    <rdfs:label rdf:datatype="http://www.w3.org/2001/XMLSchema#string">1. Skin_of_head</rdfs:label>
    <rdfs:label rdf:datatype="http://www.w3.org/2001/XMLSchema#string">2. Head_Skin</rdfs:label>
    <rdfs:label rdf:datatype="http://www.w3.org/2001/XMLSchema#string">3. Skin_structure_of_head</rdfs:label>
    <rdfs:label xml:lang="en">Head Skin</rdfs:label>
    <rdfs:label xml:lang="en">Head skin</rdfs:label>
    <rdfs:label xml:lang="en">Skin of head</rdfs:label>
    <rdfs:label xml:lang="en">Skin_structure_of_head</rdfs:label>
    <rdfs:subClassOf rdf:resource="http://merging#Code_24805"/>
    <rdfs:subClassOf rdf:resource="http://merging#Code_3840"/>
    <rdfs:subClassOf rdf:resource="http://www.ihtsdo.org/snomed#Skin_AND_subcutaneous_tissue_structure_of_head"/>
    <rdfs:subClassOf rdf:resource="http://www.ihtsdo.org/snomed#Skin_of_part_of_head_and_neck"/>
    <rdfs:subClassOf>
        <owl:Restriction>
            <owl:onProperty rdf:resource=
            "http://ncicb.nci.nih.gov/xml/owl/EVS/Thesaurus.owl#Anatomic_Structure_Is_Physical_Part_Of"/>
            <owl:someValuesFrom rdf:resource="http://merging#Code_17698"/>
        </owl:Restriction>
    </rdfs:subClassOf>
</owl:Class>
```

(b) Refactored Version

Figure B.2: Description of the Merged Class "*Code_19351*" Resulting from the *LargeBio* Integration using AROM

```
<!-- http://merging/000#Code_7845 -->

<owl:Class rdf:about="http://merging/000#Code_7845">
    <rdfs:label rdf:datatype="http://www.w3.org/2001/XMLSchema#string">1. Plane_suture</rdfs:label>
    <rdfs:label rdf:datatype="http://www.w3.org/2001/XMLSchema#string">3. Plane_suture_structure</rdfs:label>
    <rdfs:label xml:lang="en">Plane suture</rdfs:label>
    <rdfs:label xml:lang="en">Plane_suture_structure</rdfs:label>
    <rdfs:label xml:lang="lat">Sutura plana</rdfs:label>
    <rdfs:subClassOf rdf:resource="http://merging/000#Code_4280"/>
    <rdfs:subClassOf rdf:resource="http://merging/000#Code_5734"/>
    <rdfs:subClassOf rdf:resource="http://merging/000#Code_8267"/>
</owl:Class>
```

Figure B.3: Description of the Unsatisfiable Merged Class "*Code_7845*" in Example 2

```
<!-- http://merging/000#Code_20098 -->

<owl:Class rdf:about="http://merging/000#Code_20098">
    <rdfs:label rdf:datatype="http://www.w3.org/2001/XMLSchema#string">1. Apex_of_heart</rdfs:label>
    <rdfs:label rdf:datatype="http://www.w3.org/2001/XMLSchema#string">2. Apex_of_the_Heart</rdfs:label>
    <rdfs:label rdf:datatype="http://www.w3.org/2001/XMLSchema#string">3. Structure_of_apex_of_heart</rdfs:label>
    <rdfs:label xml:lang="en">Apex of heart</rdfs:label>
    <rdfs:label xml:lang="en">Apex of the Heart</rdfs:label>
    <rdfs:label xml:lang="en">Cardiac apex</rdfs:label>
    <rdfs:label xml:lang="en">Structure_of_apex_of_heart</rdfs:label>
    <rdfs:label xml:lang="lat">Apex cordis</rdfs:label>
    <rdfs:subClassOf rdf:resource="http://merging/000#Code_22984"/>
    <rdfs:subClassOf rdf:resource="http://merging/000#Code_8645"/>
    <rdfs:subClassOf rdf:resource="http://merging/001#Anatomical_point"/>
    <rdfs:subClassOf>
        <owl:Restriction>
            <owl:onProperty rdf:resource="http://merging/002#Anatomic_Structure_Is_Physical_Part_Of"/>
            <owl:someValuesFrom rdf:resource="http://merging/000#Code_29314"/>
        </owl:Restriction>
    </rdfs:subClassOf>
</owl:Class>
```

Figure B.4: Description of the Unsatisfiable Merged Class in Example 3
